# FGF21 inhibits ferroptosis caused by mitochondrial damage to promote the repair of peripheral nerve injury

**DOI:** 10.3389/fphar.2024.1358646

**Published:** 2024-09-23

**Authors:** Yao Yan, Xinyu Ran, Zihan Zhou, Yuting Gu, Rendu Wang, Chuanqi Qiu, Yinuo Sun, Jifeng Wang, Jian Xiao, Yingfeng Lu, Jian Wang

**Affiliations:** ^1^ Department of Wound Repair, The First Affiliated Hospital of Wenzhou Medical University, Wenzhou, Zhejiang, China; ^2^ Cixi Biomedical Research Institute, Wenzhou Medical University, Wenzhou, Zhejiang, China; ^3^ Wenzhou Medical University, Wenzhou, Zhejiang, China

**Keywords:** peripheral nerve injury, ferroptosis, mitochondria, fibroblast growth factor 21 (FGF21), schwann cell, ROS, lipid peroxidation

## Abstract

**Introduction:**

Ferroptosis is a new type of cell death characterized by lipid peroxidation and iron dependency, representing an emerging disease regulation mechanism. The limited understanding of ferroptosis in peripheral nerve injury (PNI) complicates the management of such injuries. Mitochondrial dysfunction, which contributes to ferroptosis, further exacerbates the challenges of peripheral nerve repair

**Methods:**

In this study, we established an in vitro model of Schwann cells model treated with TBHP and an in vivo sciatic nerve crush injury model in rats. These models were used to investigate the effects of fibroblast growth factor 21 (FGF21) on PNI, both in vitro and in vivo, and to explore the potential mechanisms linking injury-induced ferroptosis and mitochondrial dysfunction.

**Results:**

Our findings reveal that PNI triggers abnormal accumulation of lipid reactive oxygen species (ROS) and inactivates mitochondrial respiratory chain complex III, leading to mitochondrial dysfunction. This dysfunction catalyzes the oxidation of excessive polyunsaturated fatty acids, resulting in antioxidant imbalance and loss of ferroptosis suppressor protein 1 (FSP1), which drives lipid peroxidation. Additionally, irregular iron metabolism, defective mitophagy, and other factors contribute to the induction of ferroptosis. Importantly, we found that FGF21 attenuates the abnormal accumulation of lipid ROS, restores mitochondrial function, and suppresses ferroptosis, thus promoting PNI repair. Notably, glutathione peroxidase 4 (GPX4), a downstream target of nuclear factor E2-related factor 2 (Nrf2), and the ERK/Nrf2 pathway are involved in the regulation of ferroptosis by FGF21.

**Conclusion:**

FGF21 promotes peripheral nerve repair by inhibiting ferroptosis caused by mitochondrial dysfunction. Therefore, targeting mitochondria and ferroptosis represents a promising therapeutic strategy for effective PNI repair.

## 1 Introduction

Peripheral nerve injury (PNI) can result from trauma or iatrogenic intervention, leading to structural loss or functional impairment. Injuries can cause histological abnormalities such as axonal degeneration, myelin destruction, segmental demyelination, and complete Wallerian degeneration (WD) (Hewson et al., 2018). These abnormalities can result in physical disability, neuropathic pain, and reduced quality of life (Bruna Lopes et al., 2022). Despite advancements in diagnostic procedures and microsurgical techniques, functional recovery after PNI repair often remains unsatisfactory. Thus, new treatments or auxiliary strategies are needed to enhance functional recovery. Non-surgical treatments, including drug therapy, electrical therapy, and laser therapy, have been employed to promote myelination and functional recovery after PNI (Modrak et al., 2020). Compared to the central nervous system (CNS), the peripheral nervous system (PNS) possesses significant regenerative capacity. Schwann cells (SCs) play a crucial role in nerve repair, contributing to regeneration, remyelination, and axon growth (Modrak et al., 2020). Therefore, we aim to enhance the treatment of PNI through drug therapy for diseased SCs.

Ferroptosis is a regulated form of cell death characterized by the accumulation of iron-dependent lipid peroxides to lethal levels (Bersuker et al., 2019). Iron plays a crucial role in processes such as oxidative phosphorylation, myelination and metabolism (Ward et al., 2014). Lipid peroxides are produced by the peroxidation of polyunsaturated fatty acid-containing phospholipids (PUFA - PLs) in the cell membrane under conditions rich in iron and ROS. Mechanistically, eukaryotic cells are constantly attacked by lipid peroxides. If not repaired, lipid peroxides can accumulate to lethal levels, compromise the integrity of cell membranes, and ultimately trigger cell death. The morphological features of ferroptosis include unchanged nuclear size, decreased mitochondria numbers, decreased cristae, and ruptured outer membrane (Koppula et al., 2022). Several genes such as GPX4 (Li J. et al., 2020; Yang et al., 2014), FSP1 (Bersuker et al., 2019; Doll et al., 2019) and Nrf2 (Dodson et al., 2019) act as negative regulators of ferroptosis and acyl-CoA synthetase long-chain family member 4 (ACSL4) acts as a positive regulator (Huang et al., 2022). However, the research on the mechanism of ferroptosis in PNS remains limited. Recent studies have found that inhibiting ferroptosis can reduce the neuralgia caused by PNI (Guo et al., 2021), c-Jun overexpression can inhibit ferroptosis in SCs to improve PNI function (Gao et al., 2022), and HIF-1α promotes PNI recovery by enhancing the stability of SLC7A11 mRNA (An et al., 2023). Therefore, we are also interested in exploring the inhibition of ferroptosis in PNI.

The important role of cell metabolism in ferroptosis has been extensively studied. Mitochondria, are the powerhouses of cells, are crucial in maintaining cell homeostasis and function (Qiu et al., 2019). As the main regulators of oxidative phosphorylation (OXPHOS), mitochondria are the primary intracellular producers of ROS (Dan Dunn et al., 2015). Increasing experimental evidence suggests that many metabolic pathways, including cellular respiration (i.e., tricarboxylic acid cycle (TCA) and electron transport chain (ETC.), lipid metabolism and amino acid metabolism, as well as glutathione (GSH), NADPH and coenzyme Q10 (CoQ10) biosynthesis, contribute to ferroptosis through excess ROS (Gao and Jiang, 2018; Stockwell et al., 2017). Notably, iron overload induces ROS through the Fenton reaction, leading to oxidative damage and cell death. Mitochondrial iron is primarily involved in iron-sulfur (Fe-S) cluster biogenesis and heme synthesis (Conrad et al., 2018; Kowdley, 2004).

Fibroblast growth factor 21 (FGF 21), a metabolic stress hormone, is primarily secreted by the liver (Zhang et al., 2021). It plays a crucial role in glucose and lipid metabolism to maintain energy balance (Kharitonenkov et al., 2005; Leng et al., 2015). Recent studies have found that FGF21, derived from peripheral tissues, can leak into the CNS after injury and promote remyelination in a toxin-induced demyelination model (Kuroda et al., 2017). Additionally, intracellular stressors such as autophagy defects, endoplasmic reticulum stress, calcium imbalance, and mitochondrial dysfunction can induce FGF21 expression (Henkel et al., 2019; Manoli et al., 2018; Maruyama et al., 2018). Studies have shown that FGF 21 is a new oxidative stress regulator (Lu et al., 2019; Wu et al., 2021). However, the mechanism by which FGF21 regulates ferroptosis in PNI remains unclear.

In this study, we treated rat Schwann cells (RSCs) stimulated with tert-butyl hydroperoxide (TBHP) in an *in vitro* PNI model (Li B. et al., 2022; Lu et al., 2019) and Sprague-Dawley (SD) rats with sciatic nerve crush injury using FGF21. We found that FGF21 can improve mitochondrial dysfunction and ferroptosis caused by injury, thereby promoting SCs remyelination and axon regeneration. Additionally, our findings indicate that FGF21 regulates ferroptosis through the ERK/Nrf2 signaling pathway, promoting the repair of peripheral nerve injury.

## 2 Materials and methods

### 2.1 Cell culture and treatment

**FIGURE 1:**
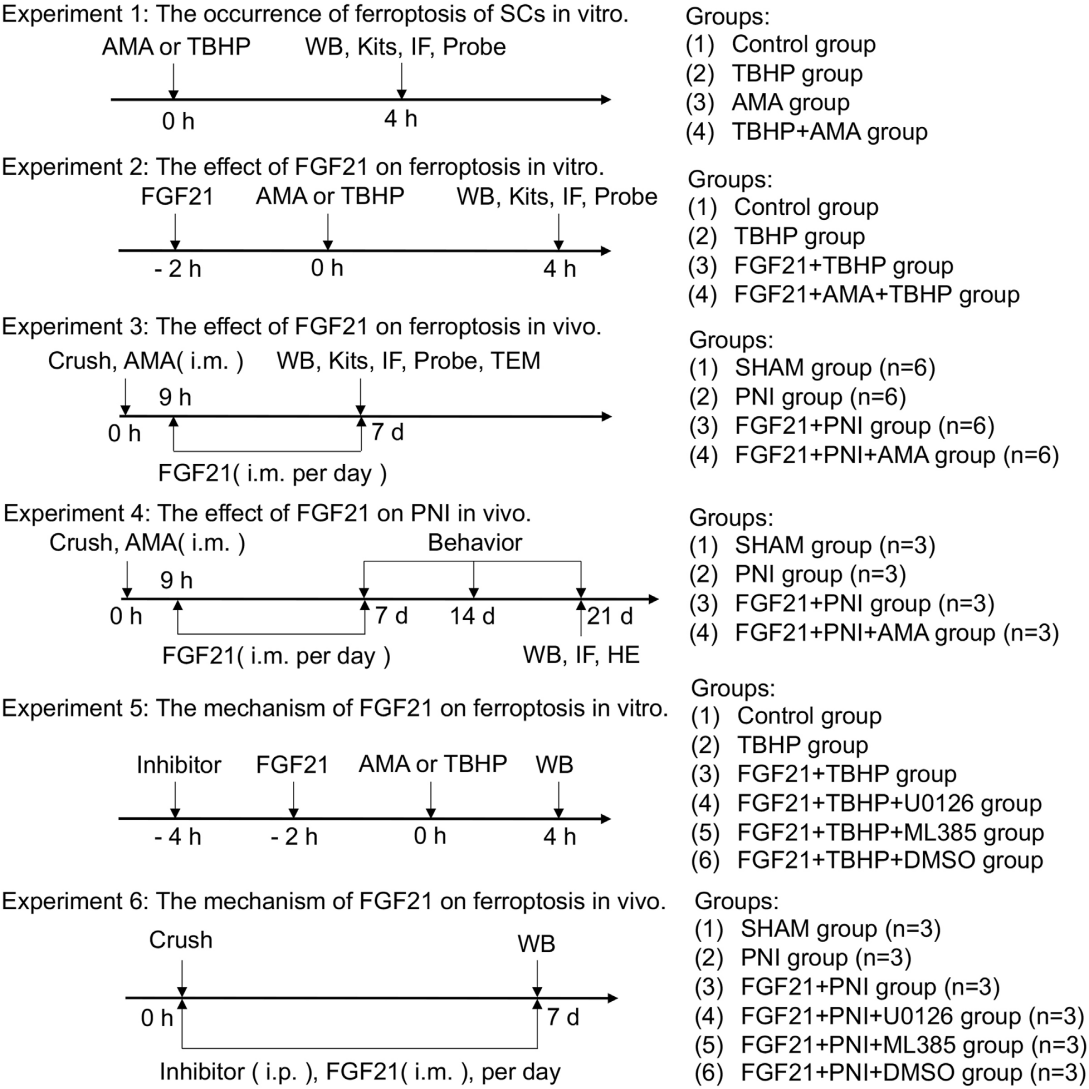
Experimental design and groups. WB: Western blot; IF: immunofluorescence; Kits: The relevant detection kit involved in the experiment; Probe: The relevant fluorescent probes are involved in the experiment; Crush: Sciatic nerve crush injury; TEM: transmission electron microscope; Behavior: walking track analysis; Inhibitor: U0126 or ML385 or DMSO; HE: Hematoxylin-eosin staining.

RSCs was obtained from the cell bank of Chinese Academy of Sciences for cell experiments. The cells were maintained in Dulbecco’s modified Eagle’s medium (DMEM) containing 10% fetal bovine serum (FBS) and 1% double antibody (penicillin and streptomycin), and cultured at 37°C in a humid incubator containing 5% CO_2_. After two passages, the cells were passaged to a 96 - well plate at a density of 8,000 cells/well for cell counting kit-8 (CCK-8) experiments. For protein extraction, the cells were seeded at an initial density of 5 × 10^5^ cells/mL in a 60 mm petri dish. In order to determine the occurrence of ferroptosis and the effect of FGF21, RSCs were divided into 6 groups: (1) Control group, (2) TBHP group, (3) AMA group, (4) AMA + TBHP group, (5) FGF21 + TBHP group and (6) FGF21 + TBHP + AMA group. The Control group was grown in normal DMEM. TBHP group was cultured in DMEM containing 100 μM TBHP for 4 h. For the FGF21 group, 250 ng/mL FGF 21 or 8 μg/mL AMA was added 2 h before TBHP was added. To further evaluate the effect of ERK/Nrf2 on ferroptosis, RSCs were pretreated with ERK inhibitor U0126 (20 μM, HY-12031) or Nrf2 inhibitor ML385 (20 μM, HY-10052) for 2 h before FGF 21 treatment (Figure 1).

### 2.2 Cell viability and drug toxicity

RSCs were seeded in 96-well plates. After the cells grew to 70%–80%, the cells were treated with drugs according to each group. The culture plates were also pre-cultured in the incubator (37°C, 5% CO_2_). After the drug treatment was completed, the cells were washed with PBS. The medium containing 10 μL CCK-8 solution/100 μL DMEM was added to each well, and then incubated for 2 h. The absorbance of each hole was measured at 450 nm using a microplate reader.

### 2.3 Quantification of ROS and lipid ROS

For *in vitro* experiments, the working solutions of mitochondrial ROS (MitoSOX, 5 µM) and dihydroethidium (DHE, 100 µM) (both from Invitrogen) were prepared according to the manufacturer’s instructions. After incubation at 37°C for 10 min, the absorption of MitoSOX and DHE at 580 nm and 610 nm was measured by fluorescence microplate reader immediately after excitation at 510 nm.

### 2.4 Mitochondrial respiratory chain complex III activity

The working solution was prepared according to the instructions (Abbkine Scientific Co., Ltd), and the mitochondrial complex was extracted from the treated RSCs according to the instructions. The absorbance at 550 nm was measured by a microplate reader at 0 min and 2 min.

### 2.5 Mitochondrial membrane potential staining (JC - 1)

JC - 1 working fluid (Beyotime Biotechnology) was configured according to the instructions. The JC - 1 working solution was added to the RSCs treatment group and incubated at 37°C for 20 min. After washing with JC-1 staining buffer (1x) for 2 times, serum - free DMEM medium was added and immediately observed under a Leica STELLARIS 5 confocal fluorescence microscope. The images were captured at a resolution of 1024 × 1024 pixels with a scan speed of 400 Hz.

### 2.6 C11 BODIPY 581/591 dyeing

The C11 BODIPY stock solution (MKbio) was prepared according to the instructions. After the cells were treated according to the requirements of each group, they were incubated at 37°C for 30 min, washed three times with PBS, and immediately observed under a Leica STELLARIS 5 confocal fluorescence microscope. The images were captured at a resolution of 1024 × 1024 pixels with a scan speed of 400 Hz.

### 2.7 ATP and MDA levels

Cells were treated as described above, and the levels of Adenosine Triphosphate (ATP, mitochondrial energy levels) and Malondialdehyde (MDA, a common product of lipid peroxidation) in the cells were assessed using the ATP and MDA assay kits (Beyotime Biotechnology), respectively, following the manufacturer’s instructions. ATP concentration was determined by the ratio of relative light units (RLU) to protein concentration. MDA concentration was obtained by the ratio of absorbance at 532 nm to protein concentration.

### 2.8 Animal models and drug therapy

Six-week-old adult male SD rats (200–220 g) were obtained from the Wenzhou Medical University. They were reared under controlled environmental conditions of temperature (about 25°C), humidity (45%–60%), and 12–12 h of light-dark cycle.

The PNI model is as described above (Li et al., 2017). The animals were anesthetized by intraperitoneal injection of two percent (v/v) pentobarbital sodium (40 mg/kg). Through the blunt separation of the biceps femoris, the right sciatic nerve was exposed horizontally in the middle of the right hind leg. Two vascular clips (large size, 6 min, Ling Bridge, China) were pressed about 2 mm apart until the sciatic nerve was transparent but not broken, and the muscle and skin were sutured. In this study, 6-week-old SD rats were randomly divided into two operation groups, PNI group and FGF21 group. The animals in the SHAM group received the same surgical procedure, but no compression was applied to the sciatic nerve. The FGF21 group received 0.25 mg/kg FGF21, which was injected intramuscularly once a day for seven consecutive days after crush injury. The SHAM group (n = 12) and the PNI group (n = 12) were given the same amount of normal saline. The FGF21 group was divided into (1) FGF21 + PNI group (n = 12), (2) FGF21 + PNI + Antimycin A (AMA) group (n = 12), (3) FGF21 + PNI + U0126 group (n = 3), (4) FGF21 + PNI + ML385 group (n = 3) and (5) FGF21 + PNI + DMSO group (n = 3). Antimycin A (AMA, sc-202467, i. m., 5 mg/kg) was administered immediately after surgery and waited for 9 h before FGF21, U0126 (HY-12031, i. p., 30 mg/kg) and ML385 (HY-10052, i. p., 30 mg/kg) were administered continuously for 7 days after surgery. On the 7th and 21st day after surgery, the animals in the group were randomly selected and sacrificed, and the right sciatic nerve was collected to evaluate the pathological indicators (Figure 1).

### 2.9 Walking track analysis

In order to evaluate the recovery of motor function, as previously mentioned, all rats were subjected to walking trajectory analysis at 1, 2, and 3 weeks after surgery (Li et al., 2017). The hind limbs of the rats were evenly dipped in black ink, and the rats were free to move forward in the black box. The changes in the position of the claw marks were recorded, and the sciatic function index (SFI) was calculated using the method proposed by Bain et al. (1989). The formula used was: SFI = 109.5 (ETS - NTS)/NTS – 38.3 (EPL - NPL)/NPL + 13.3 (EIT - NIT)/NIT - 8.8, an SFI value of 0 indicates normal sciatic nerve function, while a value of −100 indicates complete damage (Bain et al., 1989). All experiments were repeated by at least two independent researchers, and the person who performed the surgery never participated in the behavioral experiment.

### 2.10 Morphological and histological analysis

For the sciatic nerve cryosection (with at least 3 rats per group, the sciatic nerve was transected between two vascular clamps, each segment approximately 1 cm long after transection), half of the sciatic nerve, including the crush injury area, was preserved overnight in 4% paraformaldehyde at low temperature. The tissue was initially immersed in a 20% sucrose solution and maintained at a temperature of 4°C. Following natural sedimentation, it was subsequently transferred to a 30% sucrose solution and kept at the same temperature of 4°C. Subsequently, following sedimentation, the tissue was transferred to an OCT embedding medium, subsequently frozen using liquid nitrogen, and preserved at a temperature of - 80°C. The frozen-embedded tissue from day 21 was sectioned to a thickness of 10 μm, stained with hematoxylin and eosin (HE) according to standard protocols, and photographed under an optical microscope. Concurrently, the remaining 10 μm sections from day 21 were used for immunofluorescence staining.

For transmission electron microscopy (TEM), the sciatic nerve containing the compression site was dissected from the rats in each group from day 7. A 2 mm segment of the sciatic nerve, located within 1 cm of the compression end, was immediately placed in 2.5% glutaraldehyde fixation solution at 4°C. The remaining sciatic nerves from each group were stored at −80°C for protein extraction or kit measurements. The fixed sample was then handed over to the electron microscopy staff for subsequent dehydration, embedding, sectioning, and electron staining. The prepared sample sections were then observed under TEM to examine the morphology of the mitochondria in the sciatic nerve.

### 2.11 Quantification of lipid ROS in neurons

For *in vivo* experiments, fresh sciatic nerves were taken on the 7th day after the completion of the animal model. Each group included at least three rats. After nerve isolation, the sciatic nerve on the affected side (about 2 cm) was cut between two vascular clamps. One half was fixed in paraformaldehyde for frozen embedding, while the other half was embedded directly in optimal cutting temperature (OCT) compound without any fixation. Section the unfixed tissue embedded in OCT into 10 µm thick slices, and immediately incubate with MitoSOX at 37°C for 10 min. After washing with PBS buffer, stain with DAPI, mount the coverslip, and proceed with confocal imaging immediately. The images were captured using a Leica STELLARIS 5 confocal fluorescence microscope, with a resolution of 1024 × 1024 pixels, and a scan speed of 400 Hz.

### 2.12 GSH levels

Cells were processed and sciatic nerves were collected as described above, and the levels of total GSH in cells and sciatic nerves were assessed using the GSH assay kit (Beyotime Biotechnology) according to the manufacturer’s instructions. GSH concentration was obtained by the ratio of absorbance at 412 nm to protein concentration.

### 2.13 Iron assay kit

The working solution was prepared according to the instructions (DOJINDO), and the absorbance of the sciatic nerve sample at 593 nm was measured by a microplate reader.

### 2.14 Immunofluorescence staining

The treated cells were fixed in 4% paraformaldehyde for 20 min at room temperature, or frozen sections with a thickness of 10 μm were balanced at room temperature for 30 min, washed with PBS to remove the embedding agent, and incubated in PBS buffer containing 0.1% Triton X100% and 5% BSA at 37°C for 1 h. GPX4 (1:200, Abcam, ab125066), TOM20 (1:200, Proteintech, 66777-1-Ig), MBP (1:200, Cell Signaling Technology, 78896S), NF200 (1:200, Cell Signaling Technology, 2,836) were incubated overnight at 4°C, washed with PBS for 7 min * 3, and then incubated with Alexa - Fluor 488 donkey anti - rabbit IgG (1:1,000, Abcam, ab150073). And Alexa-Fluor 647 donkey anti - mouse IgG (1:1,000, Abcam, ab150108) was incubated at 37°C for 1 h. After washing with PBS for 7 min * 3, the slides were mounted with DAPI containing a fluorescence quencher. All images were captured under a Leica STELLARIS 5 confocal microscope. Excitation was achieved using 405 nm, 488 nm and 647 nm laser lines. The images were captured at a resolution of 1024 × 1024 pixels with a scan speed of 400 Hz.

### 2.15 Western blotting analysis

In order to extract the total tissue protein, the contusion center (about 2 cm long) of the sciatic nerve segment and the cells treated in each group were lysed in BOSTER Biological Technology containing phosphatase inhibitor (10 μL/mL, Applygen Technologies Inc.) and protease inhibitor PMSF (10 μL/mL, Beyotime Biotechnology). Centrifuge at 12,000 rpm/min for 20 min at 4°C. Finally, the supernatant was collected for protein determination. The above extracts were quantified using Carmassi Bradford Reagent (Thermo, Rockford, IL, United States). 30 μg of protein was resolved by SDS-PAGE gel and transferred to PVDF membranes (Bio-Rad, Hercules, CA, United States). The membrane was blocked in 5% milk containing 0.01% Tween 20 in TBS for 1 hour and incubated overnight at 4°C with the following primary antibody solutions diluted to the corresponding concentrations (Beyotime Biotechnology): S100β (1:1,000, Abcam, ab52642), GPX4 (1:2,000, Abcam, ab125066), GFAP (1:300, Cell Signaling Technology, 12,389), MBP (1:1,000, Cell Signaling Technology, 78,896), FSP1 (1:1,000, Proteintech, 20886-1-AP), FTH (1:1,000, Affinity, DF6278), ACSL4 (1:1,000, ABclonal, A6826), UQCRC1 (1:1,000, Proteintech, 21705-1-AP), HO-1 (1:1,000, Proteintech, 10701-1-AP), SOD2 (1:1,000, Proteintech, 24127-1-AP), NQO-1 (1:1,000, Proteintech, 67240-1-Ig), p62 (1:1,000, Proteintech, 66184-1-Ig), Parkin (1:500, ABclonal, A0968), Nrf2 (1:2000, Affinity, AF0639), ERK (1:2000, Cell Signaling Technology, 4,695), p-ERK (1:2,000, Cell Signaling Technology, 4,370) and GAPDH (1:1,000, ZEN-BIOSCIENCE). The membrane was washed three times with TBST and incubated with horseradish peroxidase-conjugated anti-rabbit or anti-mouse secondary antibodies at room temperature for 1 h. The ChemiDocXRS + imaging system (Bio-Rad Laboratories, Hercules, CA, United States) was used to visualize the signal and quantify the band density. The above experiments were repeated three times.

### 2.16 Statistical analysis

Data are expressed as individual points and means or as mean ± SEM. GraphPad Prism 9 software was used for statistical analysis. The 2-tailed t-test, 1-way ANOVA, 2-way ANOVA, 3-way ANOVA and Dunnett multiple comparison test were used for the statistical analysis. P ≤ 0.05 was considered statistically significant (*P < 0.05, **P < 0.01, ***P < 0.001 and ****P < 0.0001).

### 2.17 Study approval

The Animal Use and Care Program complies with the National Institutes of Health guidelines for the care and use of laboratory animals. All animal experiments were approved by the Animal Management and Use Committee of the Animal Experiment Center of the Animal Care and Use Committee of Wenzhou Medical University license to No. SCXK [ZJ] 2024-0001.

### 2.18 Data availability

Images are available from the corresponding author upon request. All data points in graphs are reported in the Supporting Data Values file.

## 3 Results

### 3.1 TBHP can induce mitochondrial respiratory chain damage and lead to mitochondrial dysfunction of RSCs *in vitro*

**FIGURE 2:**
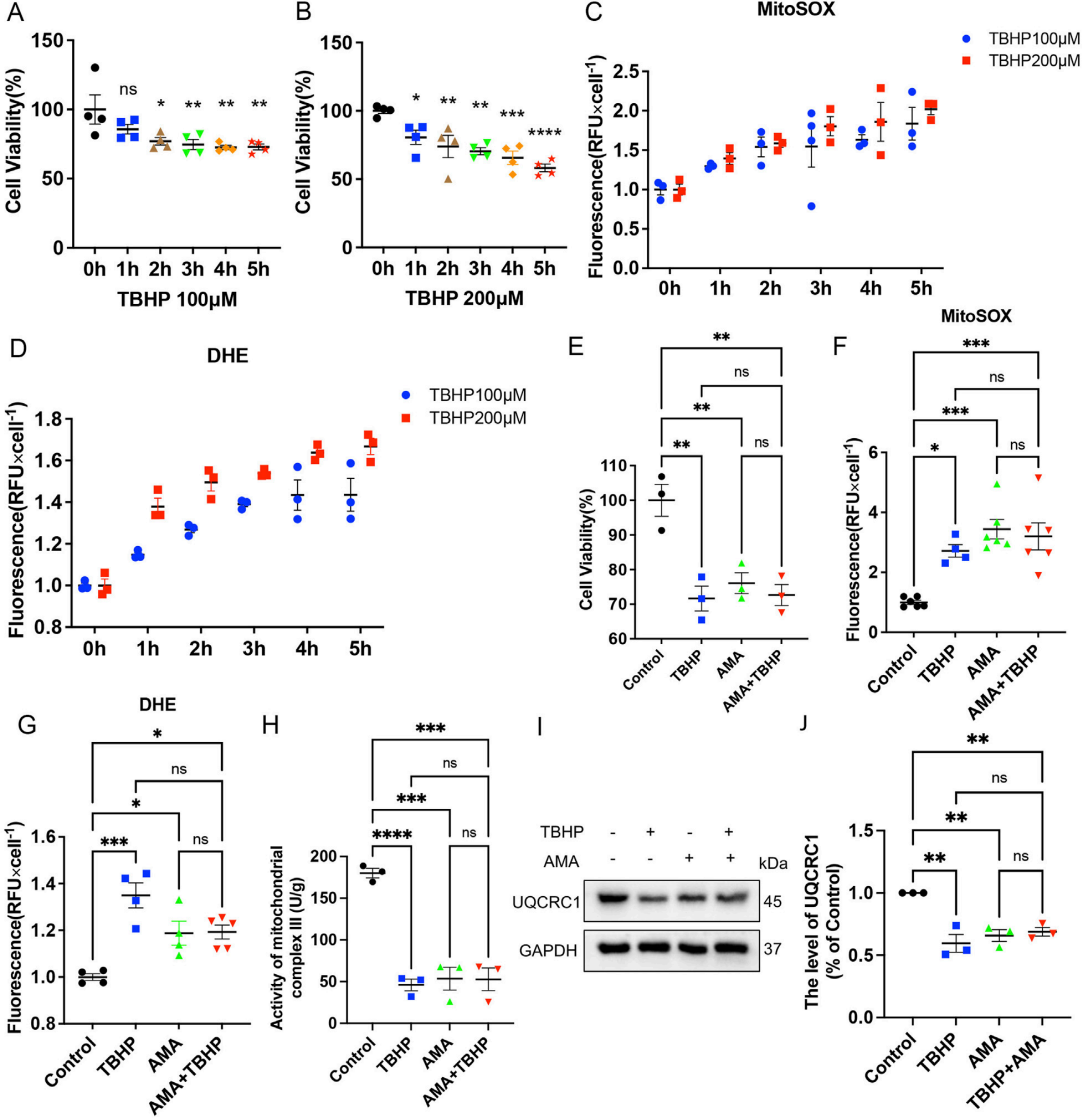
TBHP is similar to Antimycin A (AMA) in the ability to disrupt mitochondrial respiratory chain. **(A,B)** When the working concentrations of TBHP were 100 μM and 200 μM, the RSCs survived at 0–5 h, respectively. **(C,D)** When the working concentration of TBHP were 100 μM and 200 μM, the levels of MitoSOX and DHE were detected at 0–5 h. **(E)** The survival level of RSCs treated with TBHP 100 μM and AMA 8 μg/mL alone and in combination. **(F,G)** The levels of MitoSOX and DHE produced by TBHP 100 μM and AMA 8 μg/mL alone and in combination. **(H)** The activity of mitochondrial respiratory chain complex III when TBHP 100 μM and AMA 8 μg/mL acted alone and in combination. **(I)** Western blot analysis of UQCRC1, a member protein of mitochondrial respiratory chain complex III, when TBHP 100 μM and AMA 8 μg/mL acted alone and together. **(J)** Gray value analysis of UQCRC1 protein. Data were presented as mean ± SEM; n = 3–6; ns is not significant.

Previous studies have shown that PNI can lead to the occurrence of ferroptosis (Gao et al., 2022; Huang et al., 2022). To explore the mechanism of ferroptosis in this context, we first verified it *in vitro* using RSCs. SCs are the myelin sheath cells that envelop the peripheral nerve (Monk et al., 2015). To observe the effect of ferroptosis *in vitro*, we used TBHP to stimulate RSCs. Based on the statistical analysis of cell viability (Figures 2A, B), we computed the levels of MitoSOX and DHE, observing a concomitant increase in the production of these markers with the escalating concentrations and protracted exposure to TBHP (Figures 2C, D). This suggests that the generation of mitochondrial ROS and the extent of cellular oxidation are contingent upon both the duration of exposure and the concentration of the insult. To explore whether TBHP-induced lipid ROS accumulation is related to mitochondrial respiratory chain damage, we added antimycin A (AMA), a targeted inhibitor of mitochondrial respiratory chain complex III, as a comparison. We found that the cytotoxicity of TBHP at 100 μM was similar to that of AMA at 8 μg/mL, with comparable and not significantly different RSCs survival rates (Figure 2E). When TBHP and AMA were used alone or in combination, both enhanced the mitochondrial ROS level and total cellular oxidation level of RSCs, with no significant difference between the effects of TBHP and AMA alone or in combination (Figures 2F, G). This suggests that TBHP may increase ROS and lipid ROS accumulation by inhibiting the mitochondrial respiratory chain similarly to AMA. To further determine the mode of action of TBHP, we measured the activity of mitochondrial respiratory chain complex III and the expression of its member protein UQCRC1 by Western blotting. The results showed that UQCRC1 expression and complex III activity decreased, with no significant difference between the single and combined use of TBHP and AMA (Figures 2H–J). Therefore, we conclude that TBHP, like AMA, increases ROS and lipid ROS accumulation by damaging mitochondrial respiratory chain complex III.

### 3.2 TBHP induces ferroptosis by damaging the mitochondrial respiratory chain and disrupting mitochondrial function in RSCs *in vitro*

**FIGURE 3:**
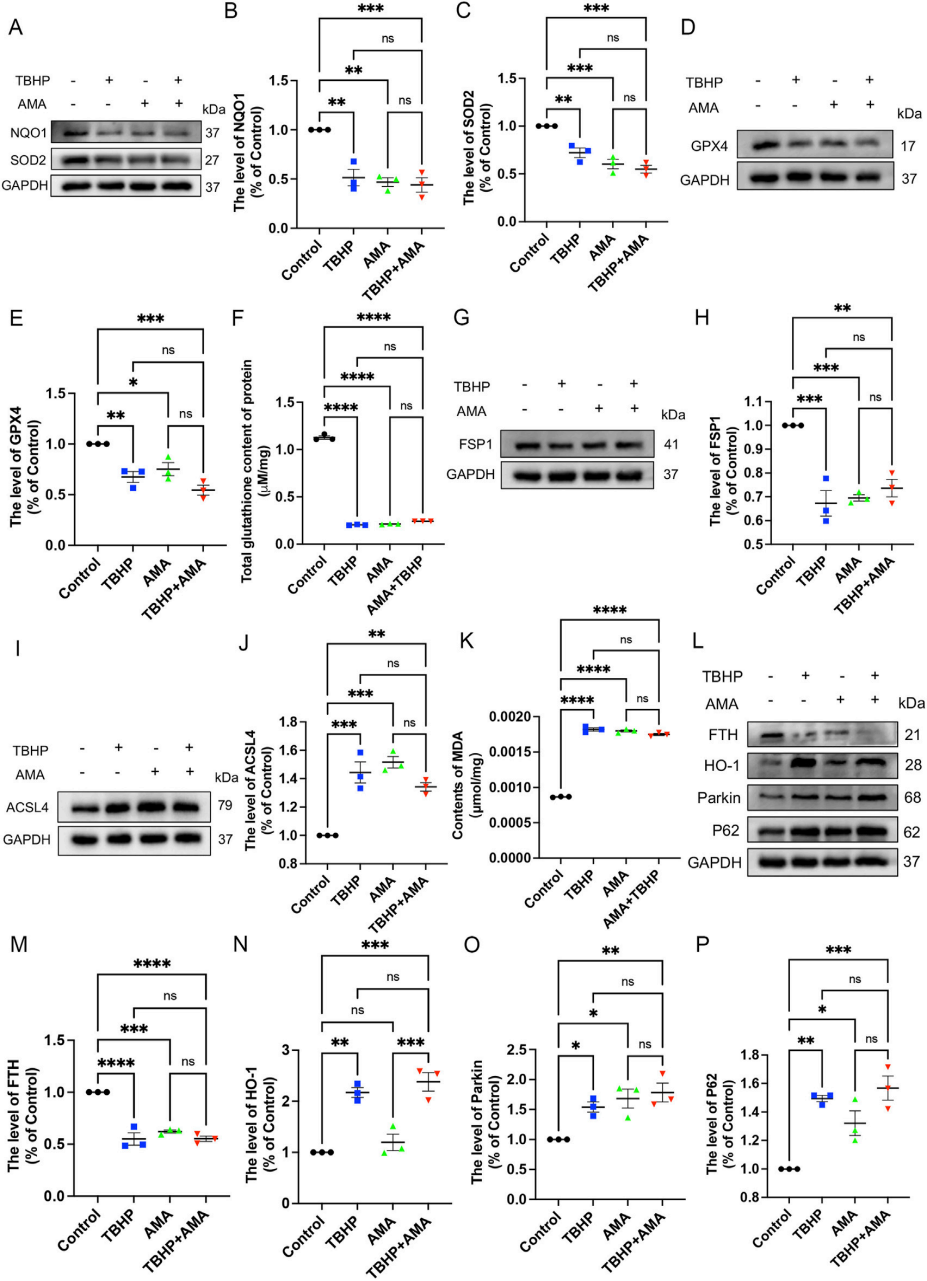
TBHP can induce RSCs ferroptosis by disrupting antioxidant and iron metabolism and disrupting autophagy patency. **(A)** Western blot analysis of NQO-1 and SOD2 in RSCs when TBHP and AMA acted alone or in combination. **(B, C)** Gray value analysis of protein NQO-1 and SOD2 expression. **(D)** Western blot analysis of GPX4 in RSCs when TBHP and AMA acted alone or in combination. **(E)** Gray value analysis of protein GPX4 expression. **(F)** The total glutathione content in each group was determined by the kit. **(G)** Western blot analysis of FSP1 in RSCs when TBHP and AMA acted alone or in combination. **(H)** Gray value analysis of protein expression of FSP1. **(I)** Western blot analysis of ACSL4 in RSCs when TBHP and AMA acted alone or in combination. **(J)** Gray value analysis of protein expression of ACSL4. **(K)** The content of MDA in each group of cells was measured by kit. **(L)** Western blot analysis of FTH, HO-1, Parkin and p62 in RSCs when TBHP and AMA acted alone or in combination. **(M–P)** Gray value analysis of protein expression of FTH, HO-1, Parkin and p62. Data were presented as mean ± SEM; n = 3; ns is not significant.

**FIGURE 4:**
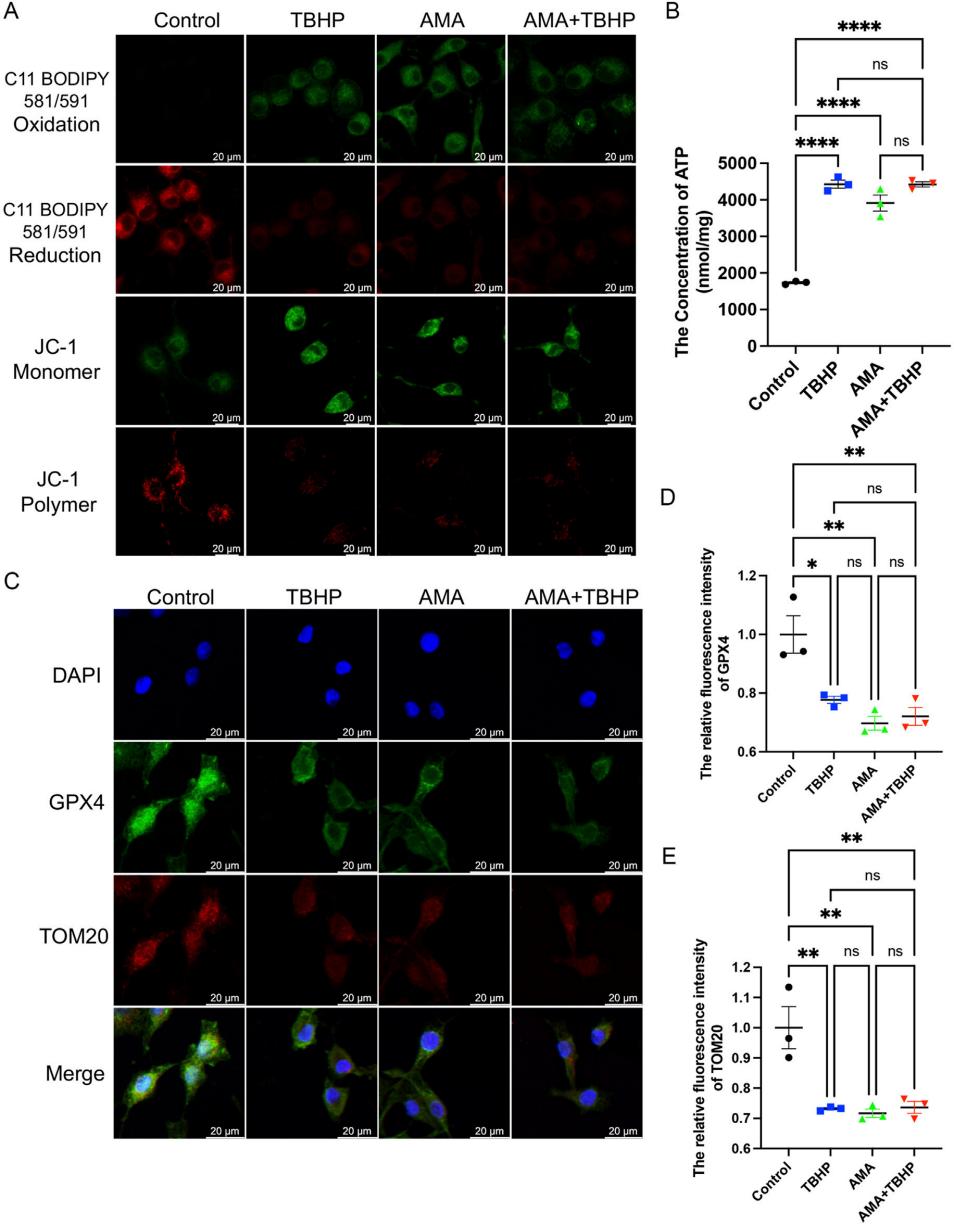
TBHP destroys mitochondria and the synthesis of glutathione, leading to lipid peroxidation-induced RSCs ferroptosis. **(A)** The fluorescence images of C11 BODIPY 581/591 and JC-1 of RSCs. Images were acquired using 20x/0.75 NA and 10x/0.4 NA objective, scale bar 20 µm. **(B)** The ATP content in each group of cells was measured using a kit. **(C)** Immunofluorescence of GPX4 and TOM20 in RSCs. Images were acquired using a 10x/0.4 NA objective, scale bar 20 µm. **(D,E)** The relative fluorescence intensity of GPX4 and TOM20 was quantified. Data were presented as mean ± SEM; n = 3; ns is not significant.

To confirm whether the accumulation of ROS and lipid ROS induced by TBHP damage to the respiratory chain indicates the sensitivity to ferroptosis and its occurrence, we assessed ferroptosis markers including antioxidant systems and lipid peroxidation. We observed that both TBHP and AMA induced oxidative stress, impaired antioxidant systems, and reduced activities of oxidoreductases such as NQO1 and SOD2 (Figures 3A–C). GPX4 was also found to be inactivated, accompanied by a decrease in GSH levels (Figures 3D–F). Inactivation of FSP1 further indicated the onset of ferroptosis and exacerbated oxidative stress (Figures 3G, H). Accumulation of ACSL4, responsible for catalyzing the oxidation of highly susceptible polyunsaturated fatty acids, and MDA, a product of lipid peroxidation, suggested lipid peroxidation occurrence (Figures 3I–K). Decreased expression of ferritin heavy chain (FTH) and increased heme oxygenase-1 (HO-1) levels indicated abnormal iron metabolism (notably, HO-1 in the AMA group did not increase, possibly due to differing ferroptotic mechanisms) (Figures 3L–N). Elevated mitochondrial autophagy (upstream autophagy, Parkin) and increased p62 downstream indicated a malignant increase in mitophagy, which can lead to ferroptosis (Figures 3L, O, P). Accumulation of lipid peroxides can be quantitatively assessed by measuring the ratio of the relative fluorescence intensity between the oxidized and reduced states when stained with C11 BODIPY 581/591. Similarly, changes in mitochondrial membrane potential can be observed by measuring the ratio of the relative fluorescence intensity between the polymerized and monomeric states when stained with JC-1 (Figure 4A) (Supplementary Figure S1). ATP content analysis revealed that TBHP and AMA led to excessive energy depletion and mitochondrial dysfunction, promoting unsaturated fatty acid synthesis and subsequent ferroptosis (Figure 4B). Immunofluorescence co-staining of GPX4 and TOM20 demonstrated mitochondrial membrane damage and reduced GPX4 expression compared to the control group (Figure 4C). Decreased overlap of GPX4 with mitochondria indicated compromised mitochondrial GPX4 expression and cytoplasmic GPX4 loss (Figures 4D, E), confirming TBHP-induced mitochondrial dysfunction and subsequent ferroptosis.

### 3.3 FGF21 improves mitochondrial function by protecting mitochondrial respiratory chain *in vitro*

**FIGURE 5:**
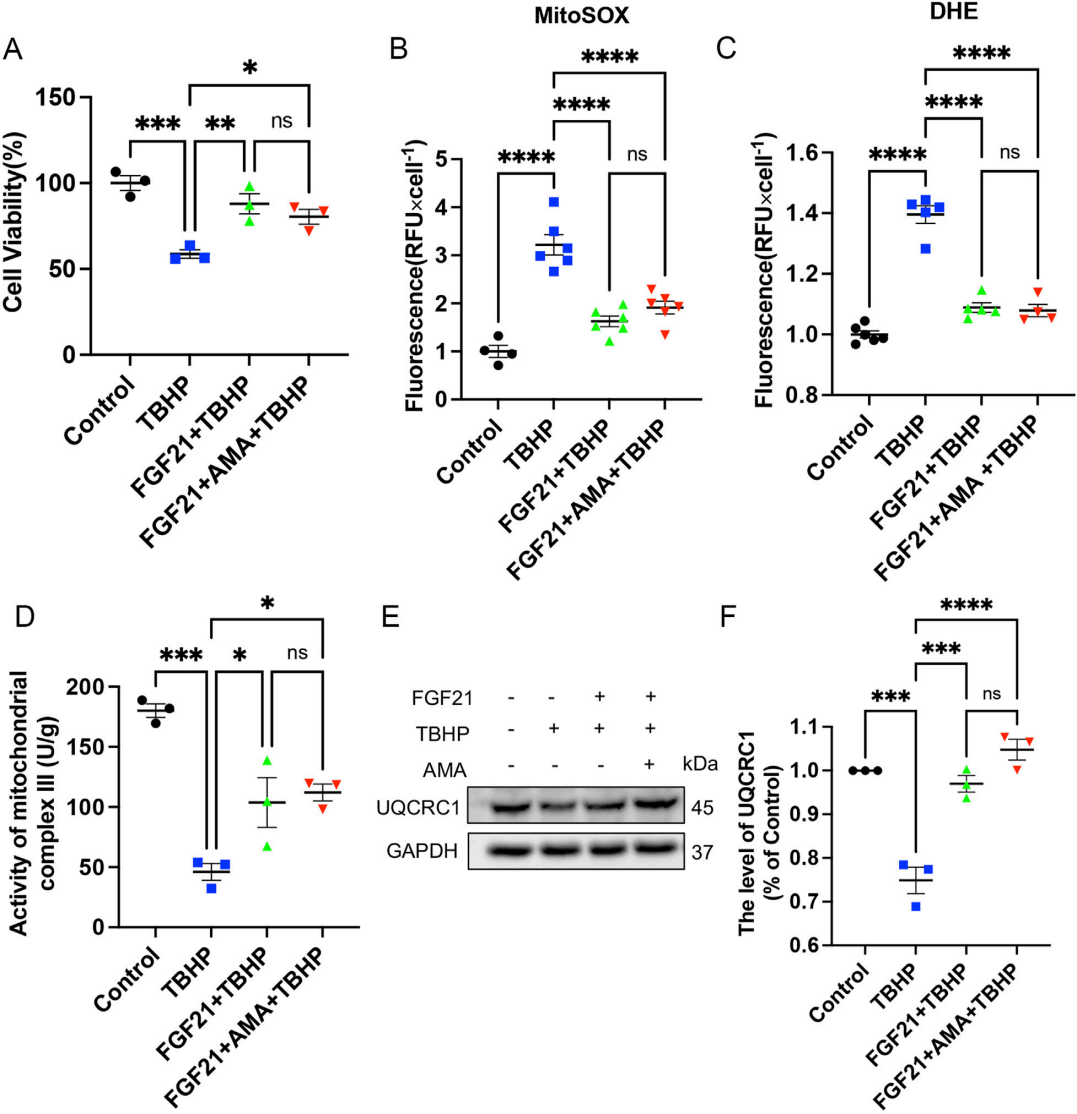
FGF21 can repair mitochondrial respiratory chain and improve mitochondrial function. **(A)** The effect of FGF21 on the survival level of RSCs when TBHP alone or TBHP combined with AMA. **(B,C)** The effect of FGF21 on the levels of MitoSOX and DHE in RSCs when TBHP alone and TBHP combined with AMA. **(D)** The effect of FGF21 on the activity of mitochondrial respiratory chain complex III in RSCs when TBHP alone or TBHP and AMA were combined. **(E)** Western blot analysis of UQCRC1, a member protein of mitochondrial respiratory chain complex III, when FGF21 acted on TBHP alone or TBHP and AMA together. **(F)** Gray value analysis of protein UQCRC1 expression. Data were presented as mean ± SEM; n = 3–6; ns is not significant.

Through *in vitro* experiments, we confirmed that PNI can induce ferroptosis, and established that mitochondrial dysfunction plays a crucial role in this process. Subsequently, we explored the regulation of ferroptosis and mitochondria. Notably, FGF21, as a novel oxidative stress regulator (Wu et al., 2021), demonstrated significant inhibition of oxidative stress at a concentration of 250 ng/mL (Lu et al., 2019). Our findings also indicate that a concentration of 250 ng/mL of FGF21 does not exhibit cytotoxicity towards RSCs, and may even facilitate cellular survival, as evidenced by preliminary experimental data (Supplementary Figure S2). Therefore, to investigate whether FGF21 also regulates ferroptosis and its mechanisms, we initially assessed levels of ROS and mitochondrial complex activity. Based on the verification of cell viability by FGF21 (Figure 5A), it was observed that FGF21 effectively regulates the accumulation of ROS and lipid ROS. This regulation results in decreased expression of MitoSOX and DHE (Figures 5B, C). Additionally, FGF21 increased the activity of mitochondrial respiratory chain complex III and upregulated expression of its constituent protein UQCRC1 compared to TBHP treatment alone (Figures 5D–F). These findings suggest that FGF21 attenuates ROS and lipid ROS accumulation, protects mitochondrial respiratory chain function, and improves overall mitochondrial function.

### 3.4 FGF21 inhibits ferroptosis by stabilizing mitochondrial function *in vitro*

**FIGURE 6:**
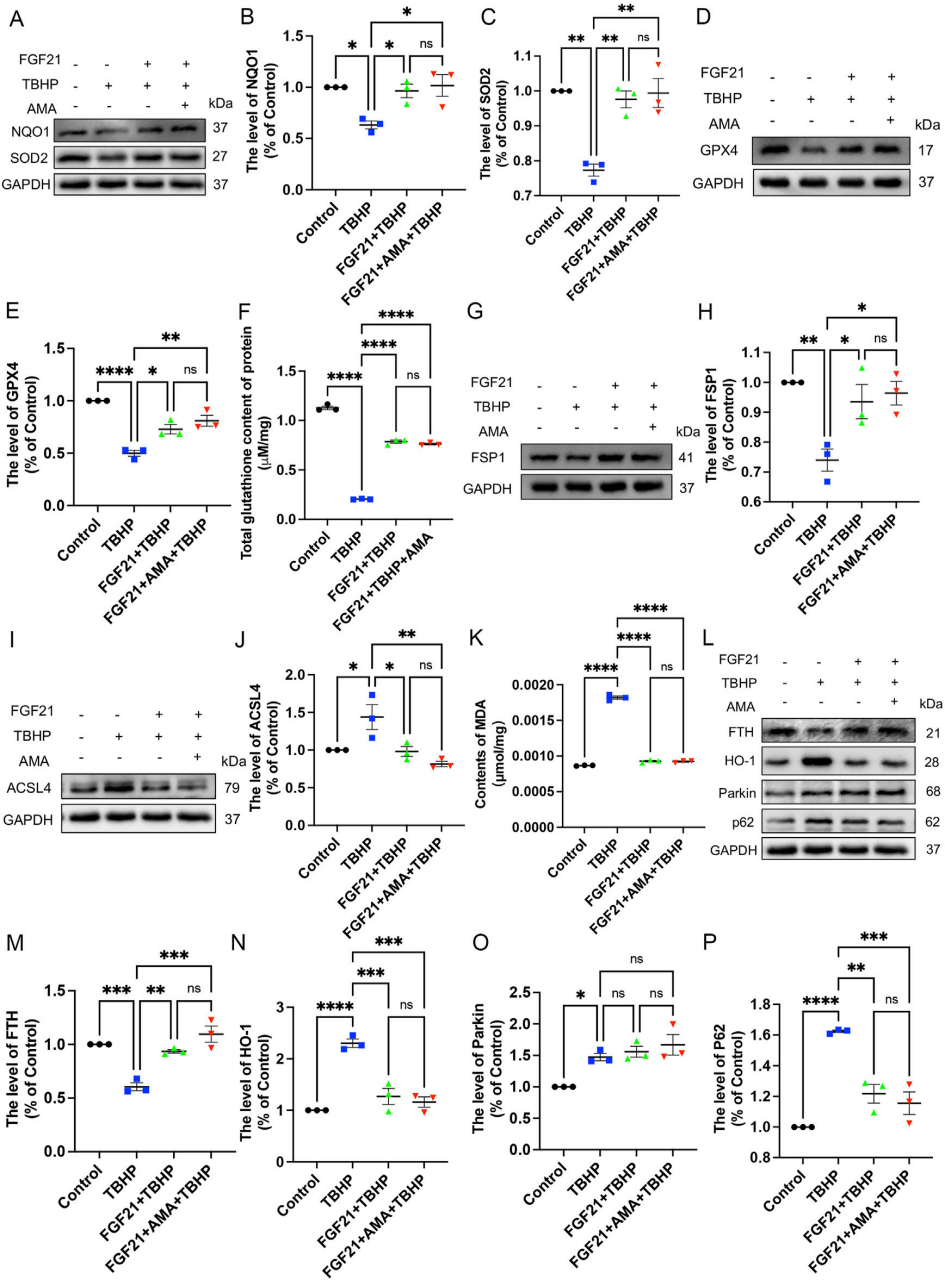
FGF21 can restore antioxidant and iron metabolism and autophagy patency to improve RSCs ferroptosis. **(A)** Western blot analysis of the effect of FGF21 on NQO-1 and SOD2 in RSCs when TBHP was alone or when TBHP and AMA were combined. **(B, C)** Gray value analysis of protein NQO-1 and SOD2 expression. **(D)** Western blot analysis of the effect of FGF21 on GPX4 in RSCs when TBHP acted alone or when TBHP and AMA acted together. **(E)** Gray value analysis of protein expression of GPX4. **(F)** The total glutathione content in each group of cells was measured using a kit. **(G)** Western blot analysis of the effect of FGF21 on FSP1 in RSCs when TBHP acted alone or when TBHP and AMA acted together. **(H)** Gray value analysis of protein expression of FSP1. **(I)** Western blot analysis of the effect of FGF21 on ACSL4 in RSCs when TBHP acted alone or when TBHP and AMA acted together. **(J)** Gray value analysis of protein expression of ACSL4. **(K)** The content of MDA in each group of cells was measured by kit. **(L)** Western blot analysis of the effect of FGF21 on FTH, HO-1, Parkin, p62 in RSCs when TBHP acted alone or when TBHP and AMA acted together. **(M–P)** Gray value analysis of protein expression of FTH, HO-1, Parkin, p62. Data were presented as mean ± SEM; n = 3; ns is not significant.

**FIGURE 7:**
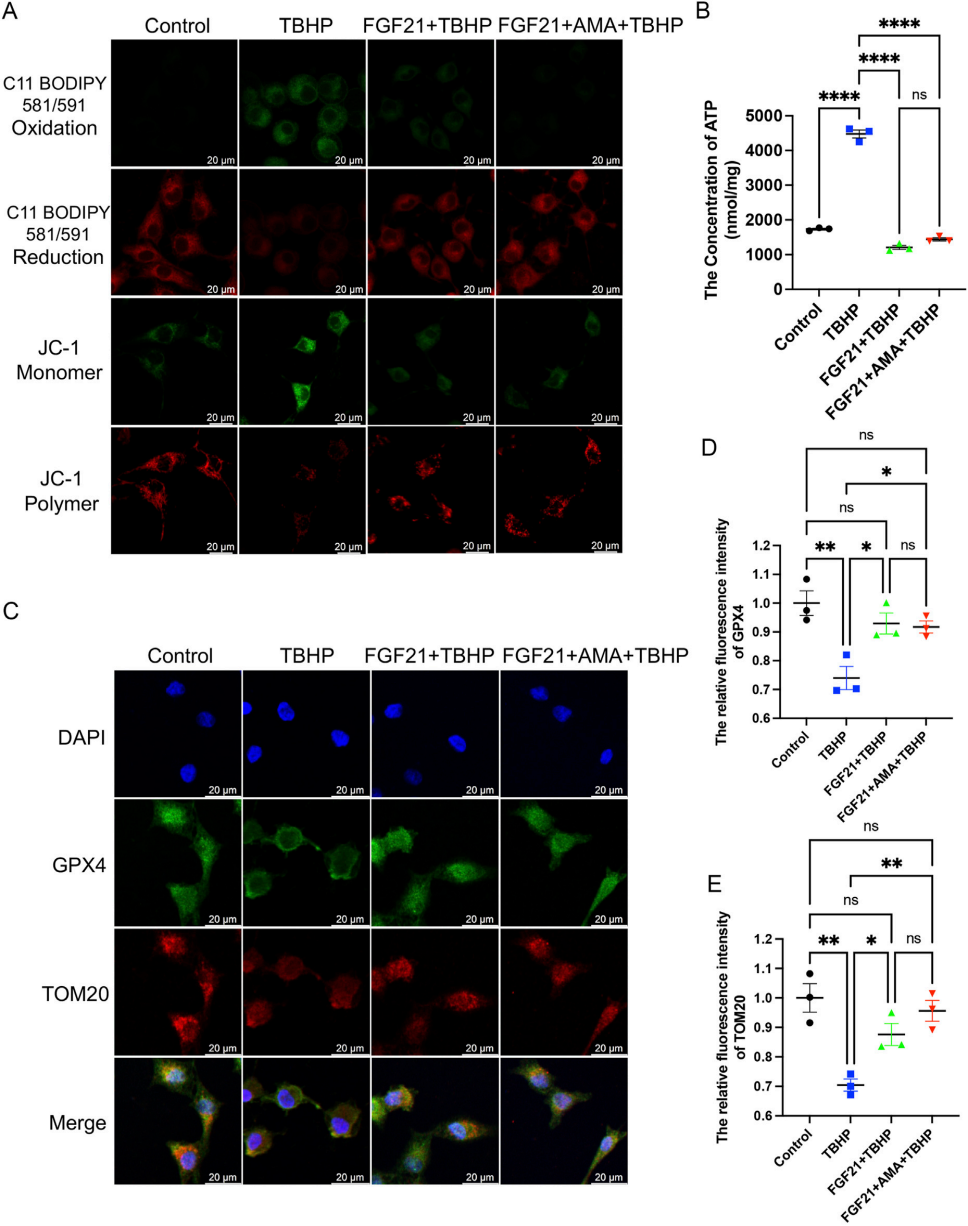
FGF21 can improve mitochondrial and glutathione synthesis to reduce lipid peroxidation and inhibit RSCs ferroptosis. **(A)** Fluorescence images of the effect of FGF21 on the C11 BODIPY 581/591 and JC-1 of RSCs. Images were acquired using 20x/0.75 NA and 10x/0.4 NA objective, scale bar 20 µm. **(B)** The ATP content in each group of cells was measured using a kit. **(C)** Immunofluorescence of the effect of FGF21 on GPX4 and TOM20 in RSCs. Images were acquired using a 10x/0.4 NA objective, scale bar 20 µm. **(D,E)** The relative fluorescence intensity of GPX4 and TOM20 was quantified. Data were presented as mean ± SEM; n = 3; ns is not significant.

Next, to further investigate the capacity and mechanism in regulating ferroptosis of FGF21, we assessed markers associated with ferroptosis. We observed that FGF21 alleviated oxidative stress compared to TBHP alone, restored the antioxidant system, and enhanced the activities of NQO1 and SOD2 oxidoreductases (Figures 6A–C). Additionally, GPX4 activity was restored, and GSH content was higher compared to the TBHP group (Figures 6D–F). Moreover, FSP1 activity was increased (Figures 6G,H), indicating inhibition of ferroptosis. The decreased expression of ACSL 4, which catalyzes the oxidation of highly oxidizable polyunsaturated fatty acids, and the reduced accumulation of lipid peroxidation-derived aldehydes like MDA, indicate an improvement of lipid peroxidation (Figures 6I–K). The increase in FTH and the decrease in HO-1 (Figures 6L–N) suggest a normalization of iron metabolism. There was no significant change in the recruitment of mitophagy marker Parkin, upstream of autophagy, suggesting mitochondrial damage may not have increased. However, the decrease in p62 downstream of autophagy suggests reduced mitophagy activity (Figures 6L, O, P). The decrease in JC-1 monomer (Green) (Figure 7A) (Supplementary Figure S2) also indicates improved mitochondrial function. Overall, FGF21 appears to enhance mitochondrial function, reduce mitochondrial damage, and affect related autophagy activities positively. Visualization using C11 BODIPY 581/591 fluorescent staining confirms the reduction in lipid peroxides (Figure 7A) (Supplementary Figure S3). ATP content detection kit results show that FGF21 improves energy homeostasis, thereby reducing unsaturated fatty acid production, restoring mitochondrial function, and inhibiting ferroptosis (Figure 7B). The immunofluorescence analysis of GPX4 and TOM20 co-staining revealed that in the FGF21-treated group compared to the Control group, there was a significant increase in the expression of TOM20 and GPX4 (Figure 7C). Particularly noteworthy was the pronounced increase in GPX4 localization around TOM20, indicating enhanced GPX4 activity both in mitochondria and cytoplasm (Figures 7D, E). These findings strongly suggest that FGF21 treatment effectively mitigates mitochondrial damage and promotes the recovery from ferroptosis.

### 3.5 FGF21 treatment can regulate mitochondrial dysfunction after peripheral nerve injury and inhibit ferroptosis *in vivo*

**FIGURE 8:**
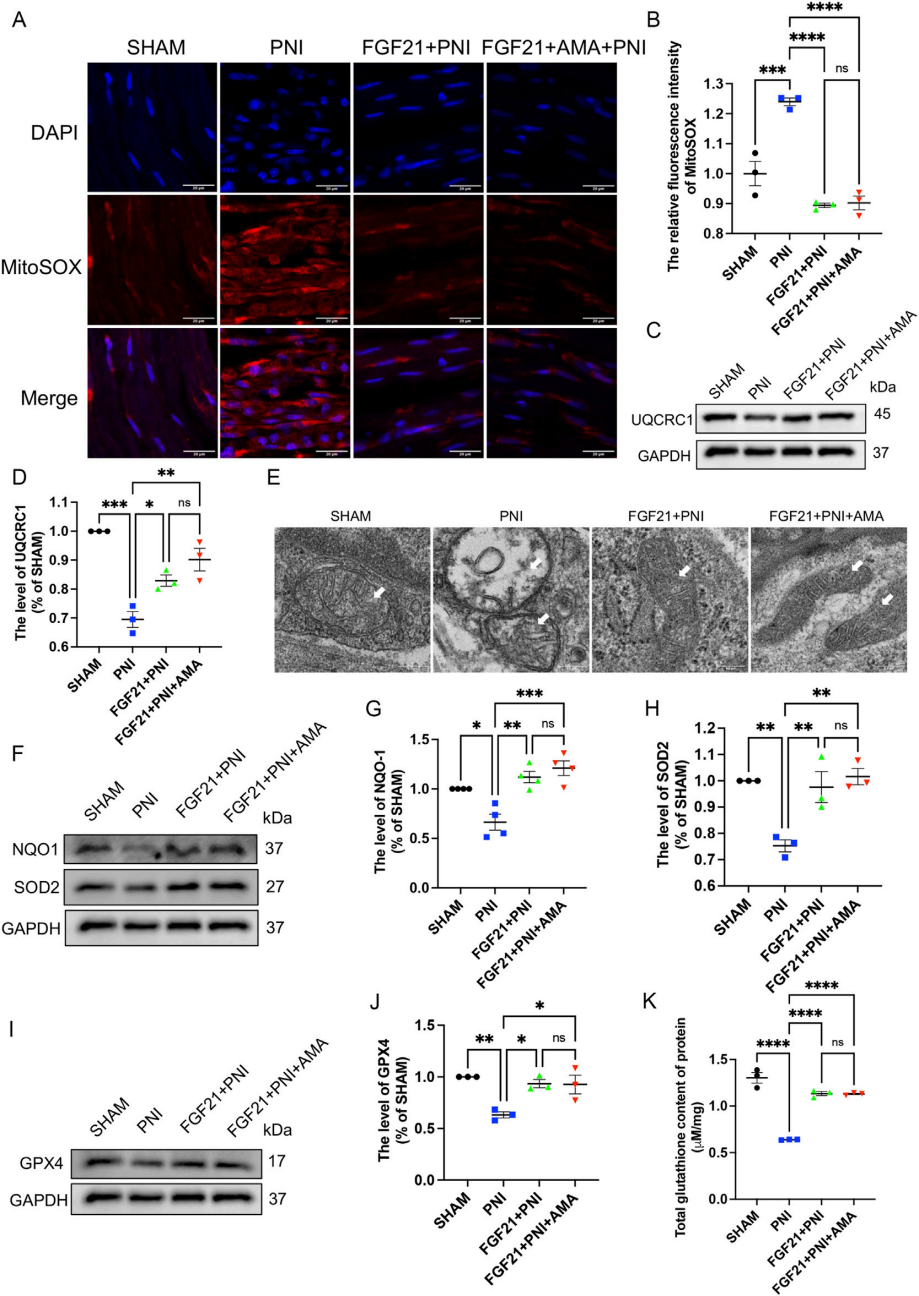
The effect of FGF21 on mitochondrial respiratory chain, antioxidant, iron metabolism and autophagic flux disorder of ferroptosis induced by sciatic nerve crush injury. **(A)** The fluorescence map of MitoSOX in the nerves of each group after FGF21 treatment was given. Images were acquired using 40x/0.95 NA objective, scale bar 20 µm. **(B)** Fluorescence expression intensity analysis of MitoSOX per unit area. **(C)** Western blot analysis of UQCRC1, a member of mitochondrial respiratory chain complex III, in the nerves of each group after FGF21 treatment. **(D)** Gray value analysis of protein UQCRC1 expression. **(E)** TEM of the sciatic nerve in each group for mitochondrial morphology, white arrows pointing to mitochondria, scale bar 0.2 μm. **(F)** Western blot analysis of proteins NQO-1, SOD2 in the nerves of each group after FGF21 treatment. **(G,H)** Gray value analysis of NQO-1, SOD2 protein expression. **(I)** Western blot analysis of proteins GPX4 in the nerves of each group after FGF21 treatment. **(J)** Gray value analysis of GPX4 protein expression. **(K)** Determination of total glutathione content in sciatic nerve of each group. Data were presented as mean ± SEM; n = 3–4; ns is not significant.

**FIGURE 9:**
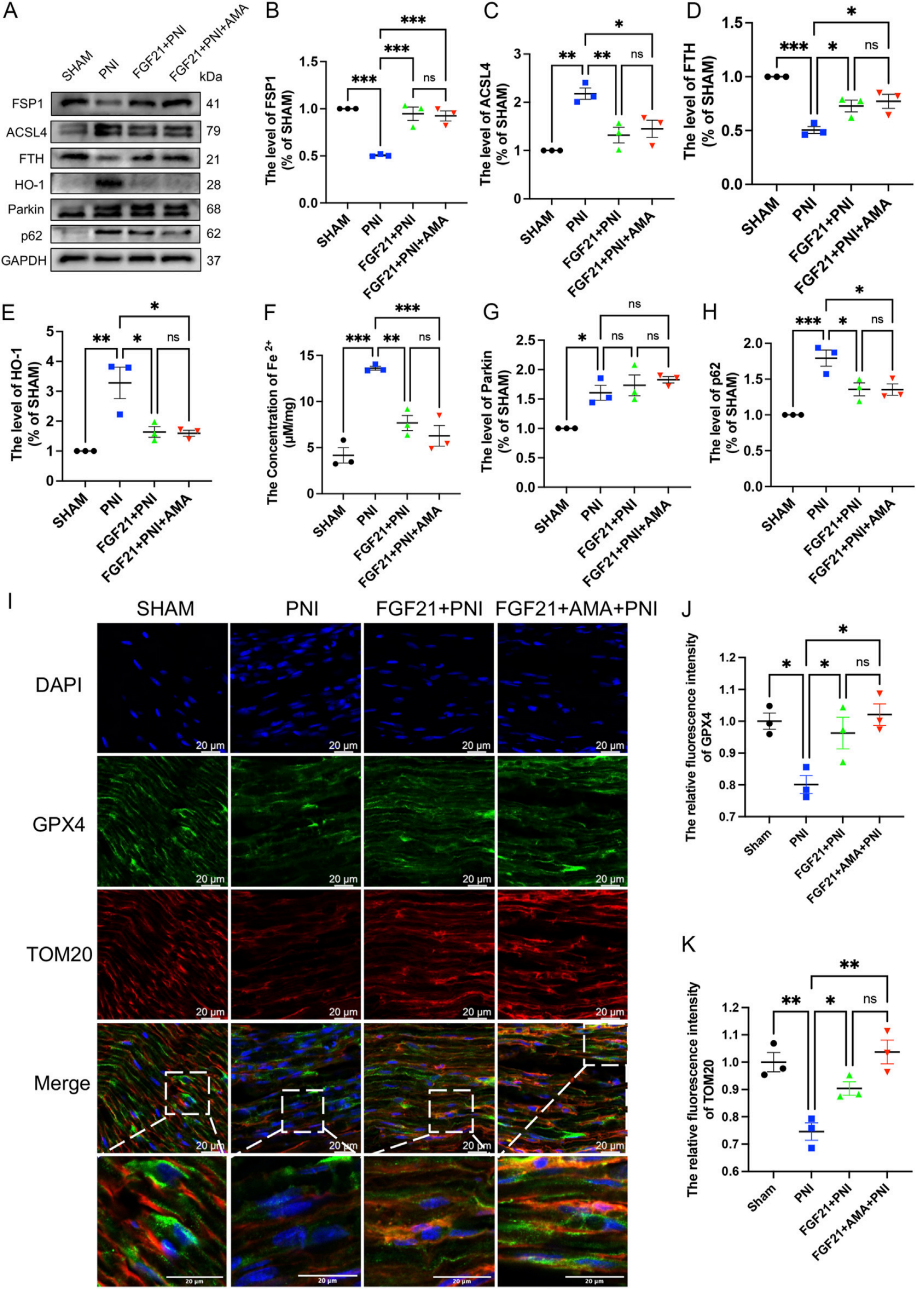
The effect of FGF21 on mitochondrial dysfunction, glutathione synthesis and abnormal accumulation of iron ions of ferroptosis induced by sciatic nerve crush injury. **(A)** Western blot analysis of proteins FSP1, ACSL4, FTH, HO-1, Parkin and p62 in the nerves of each group after FGF21 treatment. **(B–E)** Gray value analysis of protein proteins FSP1, ACSL4, FTH and HO-1 expression. **(F)** The content of Fe 2 + in each group was measured by iron colorimetric kit. **(G, H)** Gray value analysis of protein proteins Parkin and p62 expression. **(I)** Immunofluorescence of GPX4 and TOM20 in the nerves of each group after FGF21 treatment. Images were acquired using 20x/0.75 NA objective, scale bar 20 µm. **(J, K)** The relative fluorescence intensity of GPX4 and TOM20 was quantified. Data were presented as mean ± SEM; n = 3; ns is not significant.

We conducted verifications of PNI-induced ferroptosis both *in vivo* and *in vitro*, alongside evaluations of FGF21 efficacy against ferroptosis *in vitro*. The inhibitory effects of intramuscular FGF21 injection on PNI-induced mitochondrial dysfunction and ferroptosis *in vivo* were assessed. Initial findings using MitoSOX staining on fresh tissues showed that the PNI group exhibited higher levels of lipid ROS compared to the SHAM group. Remarkably, the FGF21-treated group demonstrated lower lipid ROS levels than the PNI group. (Figures 8A,B). These results suggest that intramuscular administration of FGF21 solution effectively mitigates PNI-induced mitochondrial dysfunction and ferroptosis *in vivo*. Next, to investigate whether the alteration in lipid ROS levels correlates with mitochondrial respiration, we employed Western blotting to assess the expression of URQCRC1, a member of mitochondrial complex III. Our findings revealed that the PNI group exhibited lower URQCRC1 expression compared to the SHAM group, whereas the FGF21-treated group showed higher URQCRC1 expression than the PNI group (Figures 8C, D), indicating that FGF21 can protect the mitochondrial respiratory chain *in vivo* and stabilize mitochondrial function. Then, We used TEM to capture mitochondria within the sciatic nerve. Post-PNI, a significant reduction or even disappearance of mitochondrial cristae was observed. However, treatment with FGF21 led to an increase in mitochondrial cristae (Figure 8E), affirming its role in stabilizing mitochondrial function. To further explore the potential link between mitochondrial damage *in vivo* and ferroptosis, and the therapeutic effects of FGF21, we employed Western blotting to assess antioxidant, ferroptosis-related, and autophagy-related protein levels. Our findings revealed increased activity of oxidoreductases such as NQO1 and SOD2 (Figures 8F–H), enhanced GPX4 activity (Figures 8I, J), elevated total GSH content (Figure 8K), and recovered FSP1activity (Figures 9A, B), suggesting inhibition of ferroptosis induced by sciatic nerve crush injury. Compared to the PNI group, the FGF21-treated group exhibited reduced expression of ACSL4, responsible for oxidizing highly susceptible polyunsaturated fatty acids (Figures 9A, C), indicative of improved lipid peroxidation control. Additionally, upregulation of FTH, decreased levels of HO-1, and reduced divalent iron content in nerve tissues (Figures 9A, D–F) suggested restored iron metabolism. Moreover, there was no significant change observed in the recruitment of mitophagy (Parkin, upstream of autophagy) and the decrease of p62 (downstream of autophagy) in the FGF21 group, indicating restoration of normal mitophagy levels and return to normal mitochondrial function (Figures 9A, G, H). Co-immunofluorescence analysis of GPX4 and TOM20 in frozen sections demonstrated significantly elevated expression in the FGF21 group, particularly GPX4 surrounding TOM20, highlighting the role of FGF21 in mitochondrial protection and GPX4 activity enhancement in mitochondria and cytoplasm, indicating ferroptosis recovery (Figures 9I–K). In conclusion, FGF21 effectively safeguards the mitochondrial respiratory chain *in vivo* and restores mitochondrial function, thereby inhibiting ferroptosis.

### 3.6 FGF21 treatment can improve motor dysfunction and promote myelin and axon regeneration in SD rats by inhibiting mitochondrial dysfunction and ferroptosis

**FIGURE 10:**
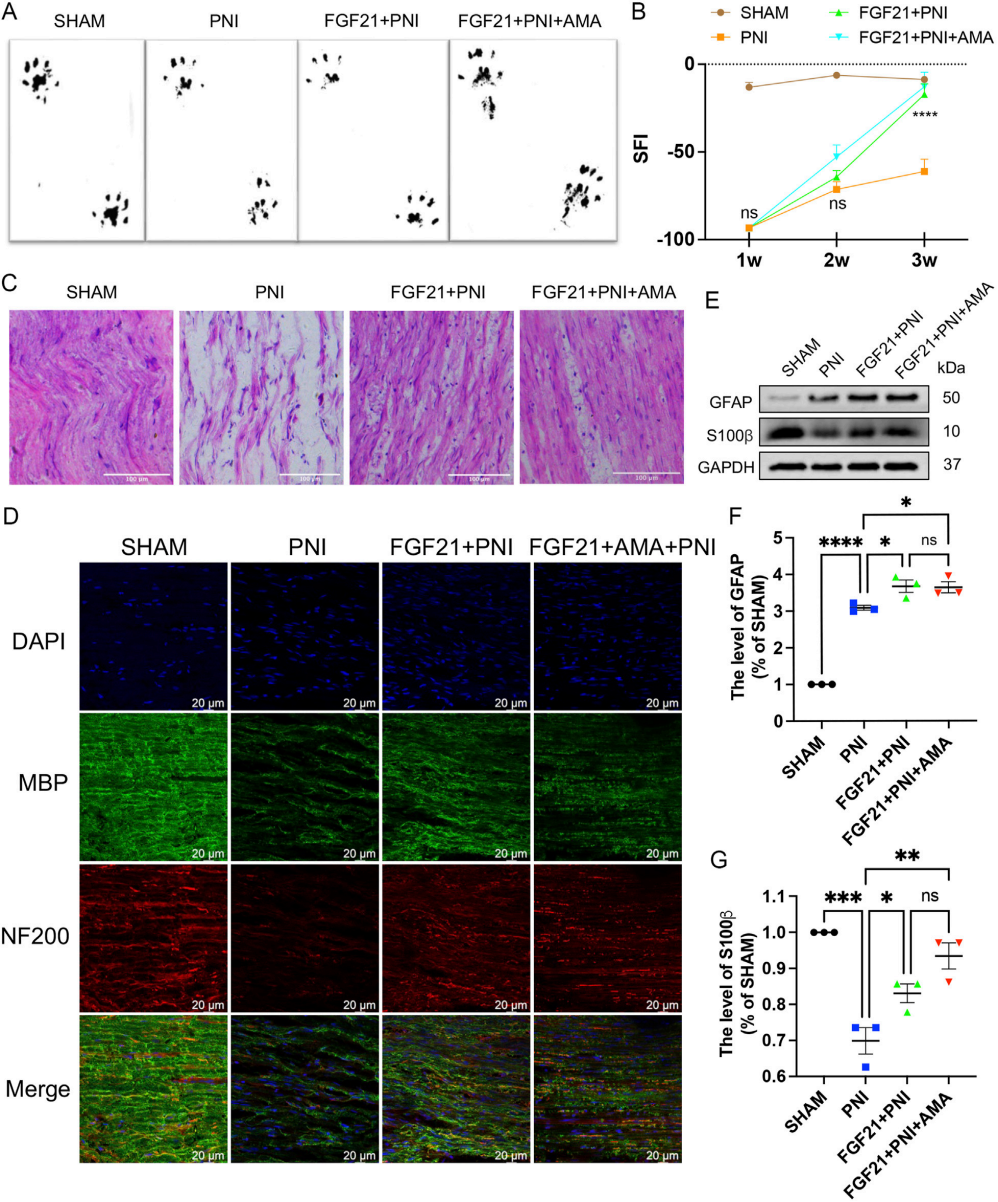
The effect of FGF21 on motor function and the repair of axons and myelin sheaths after sciatic nerve injury. **(A)** At 21 days, the hind limbs were evenly stained with black ink, and the gait imprinting of SD rats in each group. **(B)** The SFI was analyzed at 1,2 and 3 weeks in each group. **(C)** The sciatic nerve was stained with HE, and the longitudinal section of the rats in each group was observed under an optical microscope. Scale bar 100 μm. **(D)** Immunofluorescence of MBP and NF200 in the sciatic nerve of each group after FGF21 treatment. Images were acquired using 40x/0.95 NA objective, scale bar 20 µm. **(E)** Western blot analysis of GFAP and S100β in each group after FGF21 treatment. **(F,G)** Gray value analysis of protein GFAP and S100β expression. Data were presented as mean ± SEM; n = 3; ns is not significant.

To assess the efficacy of FGF 21 in enhancing recovery from PNI by inhibiting ferroptosis, we administered approximately 0.25 mg/kg of FGF21 solution continuously into the injured right hind limb for 1 week. We employed walking trajectory analysis over a 3-week period to monitor movement recovery. Initially, the SFI showed gradual improvement over time, with no significant difference observed between the PNI and FGF21 groups during the first and second weeks. However, by the third week, the SFI in the FGF21 group surpassed that of the PNI group and was comparable to the Sham group (Figures 10A, B). These results indicate that FGF21 application is beneficial for enhancing motor function recovery following crush injury.

Axonal regeneration after sciatic nerve injury is a key step in nerve recovery (Lu et al., 2019). To evaluate the histological changes of injured nerve fibers, we performed HE staining on longitudinal sections from all groups after 3 weeks of motor function recovery. Results revealed that in the FGF21 group, regenerated nerves displayed denser, more compact, and uniform newborn nerve fibers compared to the PNI group. Conversely, in the PNI group, regenerated nerve fibers appeared smaller with larger and irregular gaps between them (Figure 10C). Furthermore, to further elucidate the impact of FGF21 on myelinated nerve and axon regeneration, we conducted immunofluorescence co-staining of NF200 (heavy subunit of neurofilament, marking axons) and MBP (myelin basic protein, tracking myelin formation) on longitudinal sections. This analysis demonstrated that the FGF21 group exhibited significantly higher density and continuity of myelinated nerve fibers compared to the PNI group (Figure 10D) (Supplementary Figure S4). Collectively, these findings underscore the role of FGF21 in accelerating the regeneration of myelinated nerve fibers, as evidenced by histological improvements in nerve fiber density, size, and myelination integrity.

To investigate whether the effect of FGF21 on myelin regeneration post-PNI is associated with SCs proliferation, we proceeded to assess SCs proliferation using Western blotting. Glial fibrillary acidic protein (GFAP) is a marker of dedifferentiation or proliferation of SCs (Jessen and Mirsky, 2005), and S-100β is the main exogenous membrane protein secreted by SCs (Zhao et al., 2017). The results demonstrated that after injury, the expression of GFAP was increased in the PNI group, with even higher expression observed in the FGF21-treated group. Conversely, the expression of S-100β was significantly decreased in the PNI group but notably enhanced after FGF21 treatment (Figures 10E–G). These findings indicate that FGF21 promotes SCs proliferation following PNI, which supports myelin regeneration.

In summary, FGF21 effectively enhances motor function recovery and promotes the regeneration of myelinated nerves in SD rats following PNI.

### 3.7 FGF21 inhibits RSCs ferroptosis by regulating mitochondrial damage through ERK/Nrf2 pathway *in vitro*

**FIGURE 11:**
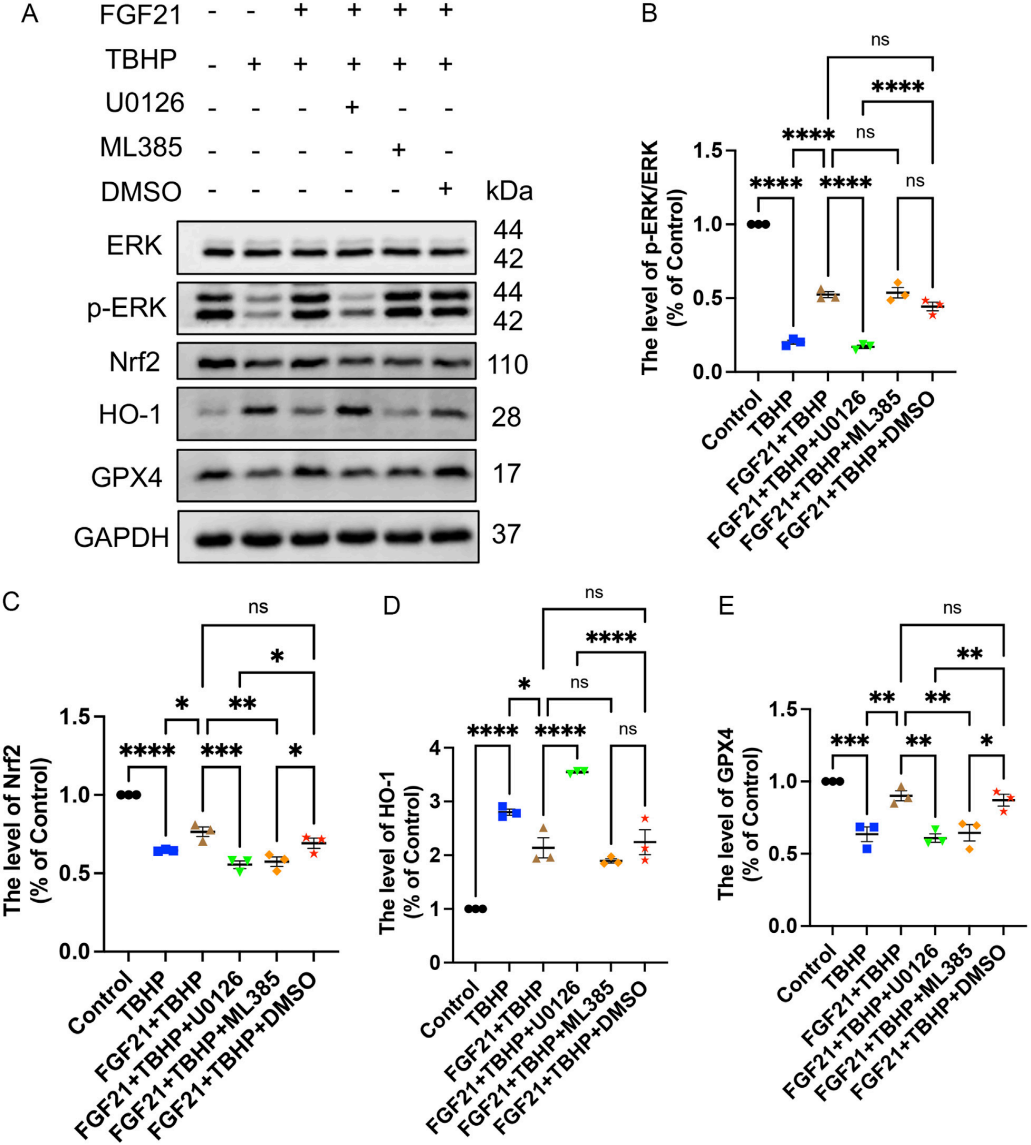
FGF21 inhibits RSCs ferroptosis by activating ERK/Nrf2 pathway to regulate mitochondrial damage *in vitro*. **(A)** When the inhibitor U0126 or ML385 was added, the effects of protein ERK, p-ERK, Nrf2, HO-1, and GPX4 were analyzed by Western blotting. **(B–E)** The expression of ERK, p-ERK, Nrf2, HO-1 and GPX4 was analyzed by gray value. Data were presented as mean ± SEM; n = 3; ns is not significant.

To investigate the regulatory mechanism of FGF21 on mitochondrial damage and ferroptosis, we conducted a series of studies. As previously reported, FGF21 protects RSCs by inhibiting oxidative damage via ERK/Nrf2 signaling (Lu et al., 2019). At the same time, a large number of studies have found that GPX4 regulates ferroptosis as a downstream of Nrf2 (Dang et al., 2022; Wang et al., 2022). To further investigate whether FGF21 can enhance antioxidant defenses and regulate mitochondrial integrity via the ERK/Nrf2 pathway to suppress ferroptosis, Western blot analysis was employed to assess the levels of ERK, p-ERK, Nrf2, HO-1, and GPX4. Our findings revealed that following FGF21 treatment, there was an increase in Nrf2 expression and the p-ERK/ERK ratio, conversely, inhibition with the ERK inhibitor U0126 or the Nrf2 inhibitor ML385 led to decreased Nrf2 expression and p-ERK/ERK ratio, respectively (Figures 11A–C). These results suggest that FGF21 exerts its anti-ferroptotic effects through the ERK/Nrf2 signaling pathway. Furthermore, we observed an increase in HO-1 expression (Figures 11A, D), which was attenuated when Nrf2 was inhibited by ML385, indicating HO-1 regulation downstream of Nrf2. Additionally, GPX4 activity was lower in the presence of either inhibitor compared to the FGF21-treated group without inhibitors (Figures 11A, E). This underscores the role of FGF21 in modulating the glutathione antioxidant system via GPX4 and regulating HO-1 activity in iron metabolism through the ERK/Nrf2 pathway, thereby stabilizing mitochondrial function and inhibiting ferroptosis.

### 3.8 FGF21 treatment stabilizes mitochondrial function by activating ERK/Nrf2 signaling pathway to inhibit ferroptosis and promote peripheral nerve injury repair *in vivo*

**FIGURE 12:**
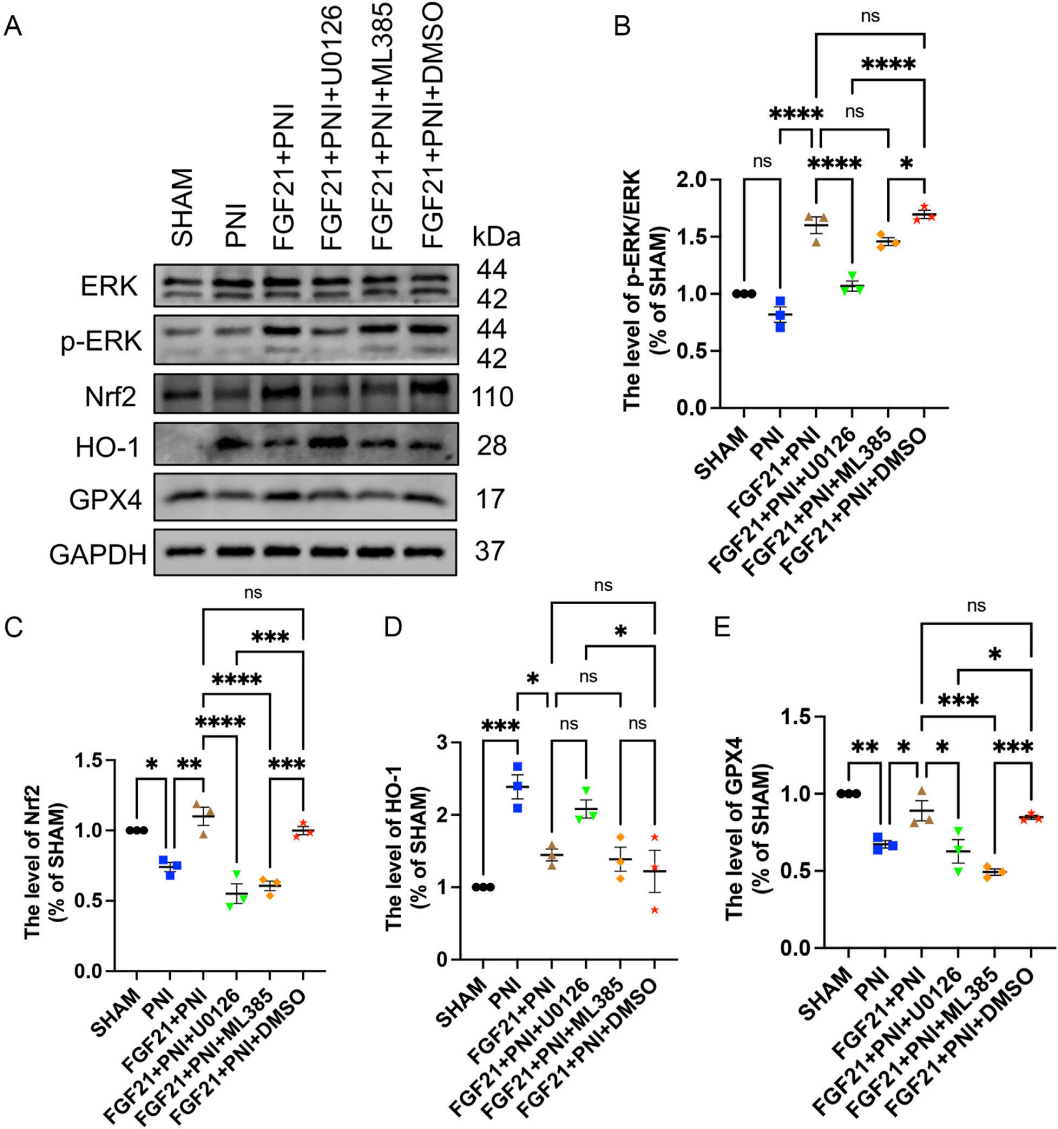
FGF21 stabilizes mitochondrial function and inhibits ferroptosis by activating ERK/Nrf2 signaling pathway *in vivo*. **(A)** The effects of ERK, p-ERK, Nrf2, HO-1 and GPX4 were analyzed by Western blotting when the inhibitors U0126 or ML385 were used respectively. **(B–E)** The expression of ERK, p-ERK, Nrf2, HO-1 and GPX4 was analyzed by gray value. Data were presented as mean ± SEM; n = 3; ns is not significant.

As previously reported, FGF21 protects RSCs by inhibiting oxidative damage via ERK/Nrf2 signaling (Lu et al., 2019), *in vitro* experiments in the previous part of this study also confirmed this statement. At the same time, a large number of studies have shown that GPX4 is involved in the regulation of ferroptosis as a downstream of Nrf2 (Dang et al., 2022; Wang et al., 2022). To investigate whether FGF21 can mitigate oxidative stress induced by sciatic nerve injury *in vivo* via the ERK/Nrf2 pathway, and subsequently regulate mitochondrial damage to inhibit ferroptosis, we employed Western blotting to assess the expression levels of ERK, p-ERK, Nrf2, HO-1, and GPX4. The results indicated that following FGF21 treatment, there was an increase in Nrf2 expression and the ratio of p-ERK/ERK, conversely, when the ERK inhibitor U0126 or the Nrf2 inhibitor ML385 was added, Nrf2 expression and the p-ERK/ERK ratio decreased (Figures 12A–C). These findings suggest that FGF21 activates the ERK/Nrf2 pathway. Compared to the FGF21 group without inhibitors, the expression of HO-1, a downstream factor of the pathway, was observed to increase (Figures 12A, D), which was attenuated when Nrf2 was inhibited by ML385, indicating HO-1 regulation downstream of Nrf2. Additionally, the activity of GPX4 was found to decrease in the presence of inhibitors (Figures 12A, E), indicating that FGF21 can regulate GPX4 and the expression of HO - 1 involved in iron metabolism through ERK/Nrf2 signaling pathway, thereby regulating mitochondrial function and inhibiting ferroptosis.

## 4 Discussion

Peripheral nerve fiber damage in the PNS can initiate WD, starting distally where axons decompose and myelin sheaths shrink. This process involves downregulation of myelin genes, dedifferentiation and proliferation of SCs, recruitment of macrophages to digest debris, and formation of a Bungner band by SCs to facilitate axon regeneration (Beirowski et al., 2005; Li R. et al., 2020; Simons and Kerschensteiner, 2014). Our study employed a sciatic nerve crush injury model to investigate ways to enhance PNI repair by accelerating injury recovery or promoting WD. However, PNI induces oxidative stress, triggering excessive ROS production that damages SCs and disrupts mitochondrial function. Mitochondrial damage, in turn, exacerbates ROS production, worsening oxidative stress and impeding PNI repair (Sayan et al., 2004). In our experiments, we used TBHP to simulate the PNI environment and sciatic nerve crush injury. Our findings revealed that PNI leads to mitochondrial ROS accumulation and impairs mitochondrial respiratory chain function. Therefore, addressing ROS accumulation to restore normal mitochondrial function emerges as a promising strategy for enhancing PNI repair.

**FIGURE 13:**
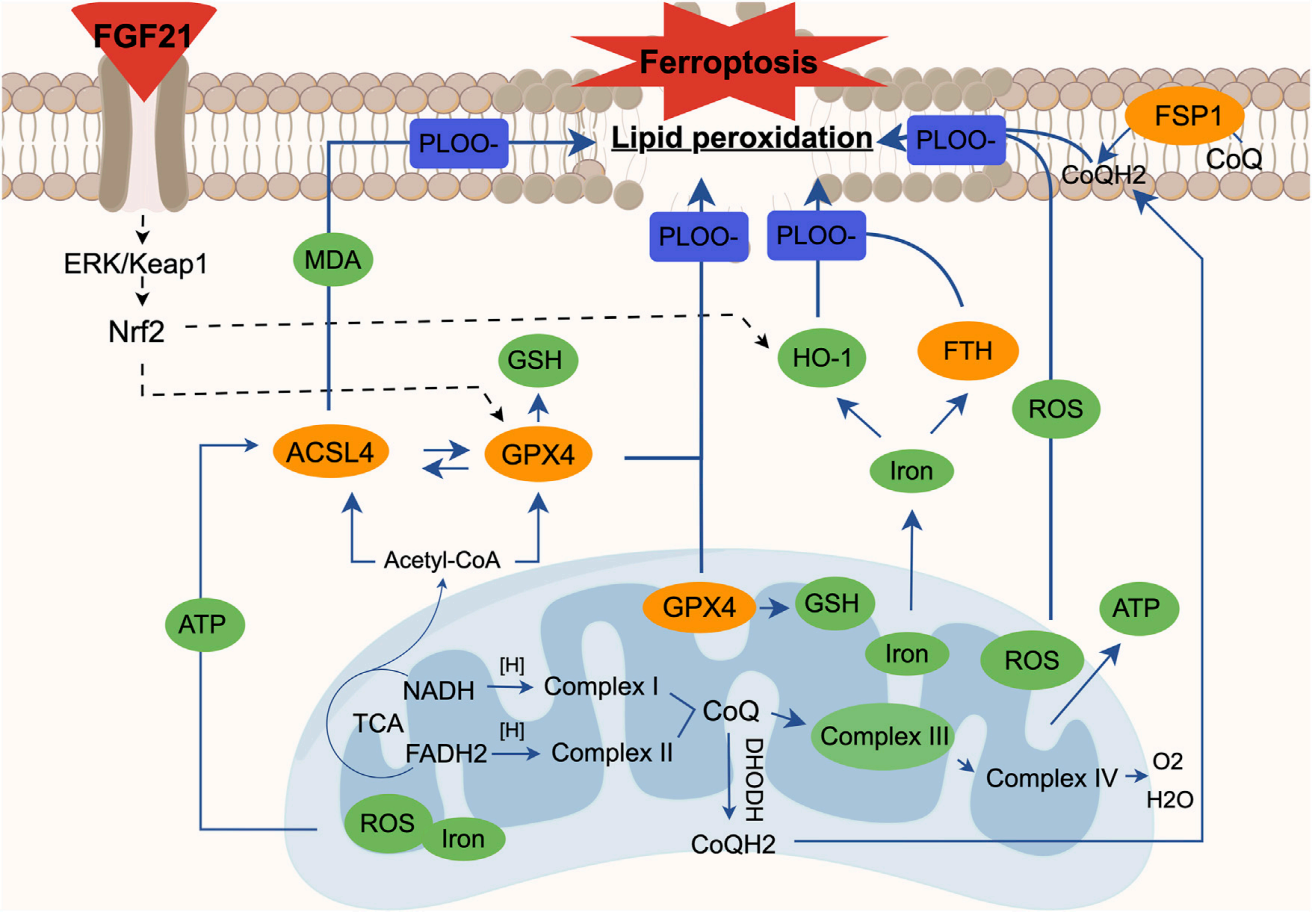
The relationship between ferroptosis and mitochondrial dysfunction. Mitochondrial ROS is essential for the occurrence of lipid peroxidation and ferroptosis, and mitochondrial electron transport and proton pump are equally important for ATP production. When the mitochondrial electron transport chain is damaged, the mitochondria as an energy field will experience abnormal accumulation of ROS and excessive ATP. At the same time, we found that the synthesis of glutathione is blocked due to the failure of glutathione peroxidase (GPX4) in mitochondria, which can cause lipid peroxidation. In the cytoplasm, due to the accumulation of unsaturated fatty acids caused by mitochondrial dysfunction, ACSL4 can easily catalyze the oxidation of unsaturated fatty acids and then derive various aldehydes such as MDA. The inactivation of GPX4 in the cytoplasm affects the synthesis of glutathione in the cell, which is not conducive to the antioxidant capacity of the cell; abnormal iron metabolism will produce more ROS through the Fenton reaction, and it will also affect the normal release of iron by heme oxygenase 1 (HO-1) and the expression of ferritins like FTH, eventually leading to lipid peroxidation. In the cell membrane, we also found a decrease in the activity of ferroptosis inhibitory protein (FSP1), which means that ubiquinone (CoQ) in the cell membrane cannot be normally converted into panthenol (CoQH2), resulting in lipid peroxidation. At the same time, it is known that when the mitochondrial function is abnormal, the imbalance between CoQ in mitochondria and CoQ in cell membrane will affect the level of lipid oxidation again. In summary, each case alone or interaction will lead to the result of cell ferroptosis. We found that FGF21 can improve the above situation. At the same time, FGF21 seems to affect the expression of heme oxygenase 1 (HO-1), mitochondrial and cytoplasmic glutathione peroxidase (GPX4) through the ERK/Nrf2 pathway, thereby hindering the process of lipid peroxidation and ultimately inhibiting cell ferroptosis.

Studies have shown a close relationship between mitochondrial metabolic activity and ferroptosis (Figure 13). Mitochondrial functions such as the TCA cycle and ETC are crucial for effective ferroptosis induction (Gao et al., 2019). Upon accumulation, mitochondrial ROS can react with PUFA in the mitochondrial membrane, leading to lipid peroxidation and damage to mitochondrial DNA (mtDNA), particularly affecting ETC complex subunits encoded by mtDNA (Barrera G et al., 2016). These phenomena are observed not only in cancer but also in conditions with heightened oxidative stress like chronic inflammation and neurodegenerative diseases, underscoring mitochondria’s involvement in ferroptosis (Battaglia et al., 2020). Pharmacologically induced ferroptosis also triggers mitochondrial ROS accumulation, mitochondrial fragmentation, changes in mitochondrial membrane potential (ΔΨm), and ATP depletion (Neitemeier et al., 2017; Yagoda et al., 2007). Furthermore, the GPX4 and FSP1 antioxidant systems play critical roles in regulating ferroptosis and interact closely with mitochondria. Studies have indicated that inhibiting autophagy can exacerbate Keap1-mediated Nrf2 degradation, further promoting ferroptosis (Li B. et al., 2022; Li Y. et al., 2022). Our findings confirme the ferroptosis in PNI, characterized by lipid peroxidation and iron dependency concurrent with mitochondrial respiratory chain damage. Comparatively, using the mitochondrial respiratory chain inhibitor AMA supports the concept that PNI damages or exacerbates mitochondrial dysfunction, thus promoting ferroptosis. In summary, ferroptosis leads to increased mitochondrial ROS, highlighting the importance of preserving mitochondrial function and mitochondrial autophagy to inhibit ferroptosis and facilitate PNI repair.

The role of FGF21 in promoting PNI repair is contentious. Research indicates that SCs express FGF21 receptors, and FGF21 levels remain low during periods of sharp myelin gene expression (Zhang et al., 2021). Consequently, there’s a hypothesis that supplementing an appropriate amount of FGF21 could potentially accelerate the expression of myelin genes, thereby expediting PNI repair. Conversely, excessive FGF21 supplementation might inhibit myelin gene expression. Nevertheless, FGF21, known for its safety and lack of toxic reactions in the body, warrants further investigation. Both previous studies and our own experiments have demonstrated that intramuscular injection of 0.25 mg/kg FGF21 for 1 week effectively promotes PNI repair.

Currently, the inhibition of ferroptosis primarily relies on the use of selective inhibitors, which poses limitations in mechanistic studies. From our research, we have observed that PNI induces oxidative stress, abnormal accumulation of mitochondrial ROS, and inhibition of mitochondrial respiratory chain, leading to mitochondrial dysfunction. FGF 21, an endocrine hormone pivotal in glucose and lipid metabolism and energy regulation (Kharitonenkov et al., 2005; Leng et al., 2015), is emerging as a novel regulator of oxidative stress in humans and rodents (Gomez-Samano et al., 2017). Studies have demonstrated that FGF21 can prevent mitochondrial dysfunction and oxidative stress in cardiomyocytes (Jin et al., 2022) and promote PNI repair by enhancing oxidative stress and mitochondrial autophagy (Lu et al., 2019; Zhang et al., 2021). Based on these findings, we hypothesize that the ability of FGF21 to ameliorate mitochondrial dysfunction may inhibit ferroptosis. While FGF21 is known to regulate cell proliferation, development, pyroptosis, apoptosis, autophagy, and mitochondrial diseases (Chen et al., 2020; Gomez-Samano et al., 2017; Salminen et al., 2017; Wu et al., 2021), few studies have investigated its role in regulating mitochondrial dysfunction and ferroptosis in PNI. In our study, using *in vivo* and *in vitro* models of PNI, we observed that FGF21 effectively reduces the accumulation of mitochondrial ROS induced by PNI and enhances the activity of mitochondrial respiratory chain complex III. This protects the mitochondrial respiratory chain, stabilizes mitochondrial function, and enhances mitochondrial antioxidant capacity by stabilizing GPX4 expression within mitochondria. Additionally, FGF21 diminishes markers of ferroptosis such as lipid peroxidation and abnormal iron aggregation, while improving the antioxidant system and mitochondrial autophagy, ultimately inhibiting ferroptosis.

Mechanistically, FGF21 stimulates extracellular signal-regulated kinase (ERK) and p38 mitogen-activated protein kinase (MAPK) in sciatic nerve cells and sciatic nerve. Studies have demonstrated that FGF21 inhibits ferroptosis-induced liver injury by activating Nrf2 and inhibiting HO-1 (Wu et al., 2021). Conversely, inhibition of the Nrf2/GPX4 axis promotes ferroptosis (Wang et al., 2022). Additionally, research has identified FSP1 as a transcriptional target of Nrf2 (Koppula et al., 2022). In cancer cells, mitochondrial ROS serve as second messengers in oncogenic signal transduction cascades, including those driven by MAPK and transcription factor NF-kB (Sabharwal and Schumacker, 2014). Through our experiments aimed at verifying the mechanism of FGF21, we have discovered that FGF21 regulates GPX4 and HO-1 via the ERK/Nrf2 signaling pathway to modulate iron metabolism, thereby stabilizing mitochondrial function and inhibiting cell ferroptosis. This ultimately contributes to repairing peripheral nerve injury (PNI) and holds promise for alleviating associated muscle atrophy. This research opens new avenues for treating PNI and addressing muscle atrophy in affected patients.

## 5 Conclusion

In general, this study shows that FGF21 can inhibit ferroptosis and repair PNI by regulating mitochondrial dysfunction induced by PNI, and it can be regulated by ERK/Nrf2 signaling pathway.

## Data Availability

The raw data supporting the conclusions of this article will be made available by the authors, without undue reservation.
