# Corrigendum: FGF21 inhibits ferroptosis caused by mitochondrial damage to promote the repair of peripheral nerve injury

**DOI:** 10.3389/fphar.2025.1584515

**Published:** 2025-06-02

**Authors:** Yao Yan, Xinyu Ran, Zihan Zhou, Yuting Gu, Rendu Wang, Chuanqi Qiu, Yinuo Sun, Jifeng Wang, Jian Xiao, Yingfeng Lu, Jian Wang

**Affiliations:** ^1^ Department of Wound Repair, The First Affiliated Hospital of Wenzhou Medical University, Wenzhou, Zhejiang, China; ^2^ Cixi Biomedical Research Institute, Wenzhou Medical University, Wenzhou, Zhejiang, China; ^3^ Wenzhou Medical University, Wenzhou, Zhejiang, China

**Keywords:** peripheral nerve injury, ferroptosis, mitochondria, fibroblast growth factor 21 (FGF21), schwann cell, ROS, lipid peroxidation

In the published article, there was an error in the **Author list**, and authors Jian Xiao and Yingfeng Lu were erroneously not included as corresponding authors. The corrected author list appears below.

Yao Yan1,2,3†, Xinyu Ran1,3†, Zihan Zhou1,3, Yuting Gu1,2,3, Rendu Wang1,3, Chuanqi Qiu1,3, Yinuo Sun1,3, Jifeng Wang1,3, Jian Xiao1,2,3*, Yingfeng Lu1,3* and Jian Wang1,2,3*

The corrected list of corresponding authors appears below: Jian Wang, jianwang0516@126.com Yingfeng Lu, lyf1101140226@163.com Jian Xiao, xfxj2000@126.com

The authors apologize for this error and state that this does not change the scientific conclusions of the article in any way. The original article has been updated.

