# Supplementary material for: FGF21 inhibits ferroptosis caused by mitochondrial damage to promote the repair of peripheral nerve injury
Source: Front Pharmacol. 2024 Sep 23;15:1358646. doi: 10.3389/fphar.2024.1358646 (PMC11456482; doi:10.3389/fphar.2024.1358646)
Supplement: Supplementary file 1 [file DataSheet1.PDF]

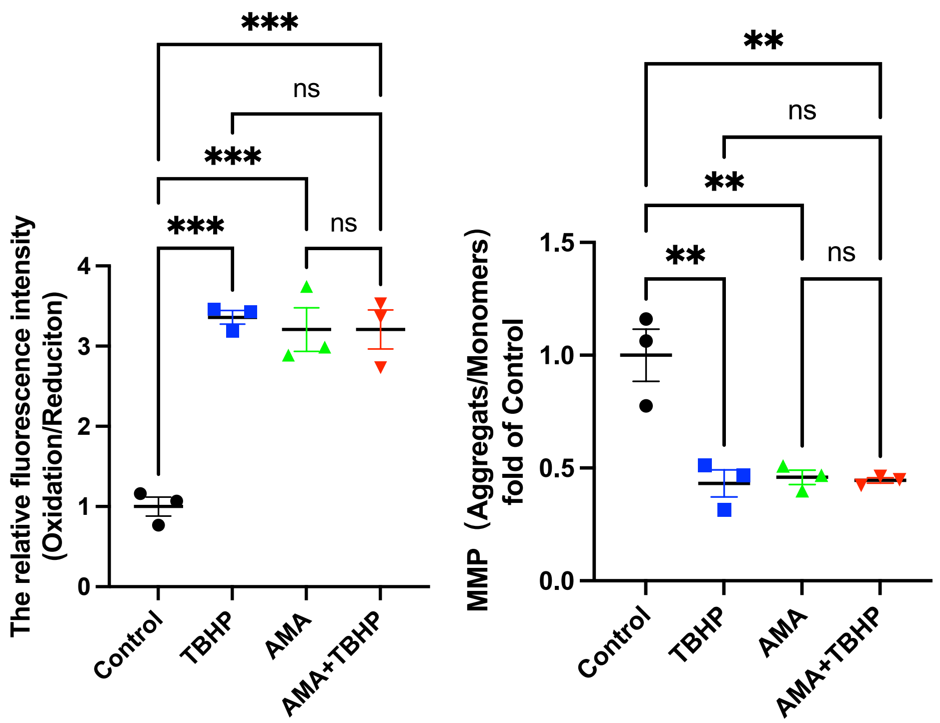

S Fig. 1 The fluorescence intensity of C11 BODIPY 581/591 and JC-1 in RSCs per unit area respectively.

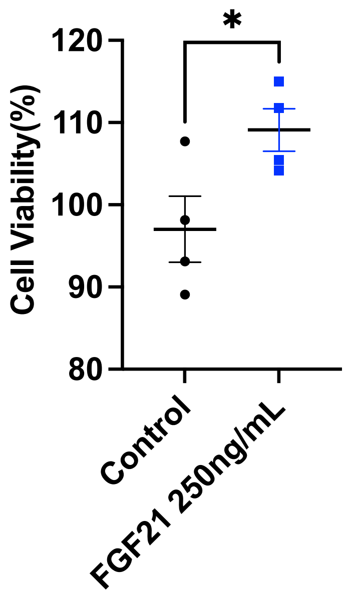

S Fig. 2 The cell viability of RSC after incubation with 250 ng / mL FGF21 for 2 hours.

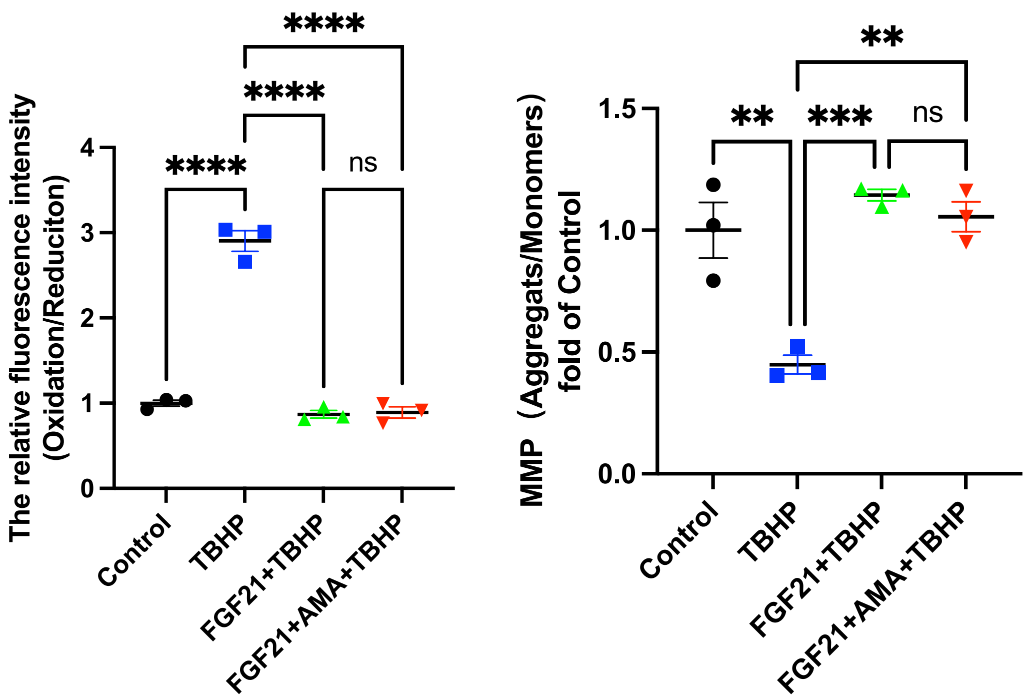

S Fig. 3 The fluorescence intensity of C11 BODIPY 581/591 and JC-1 in RSCs per unit area respectively.

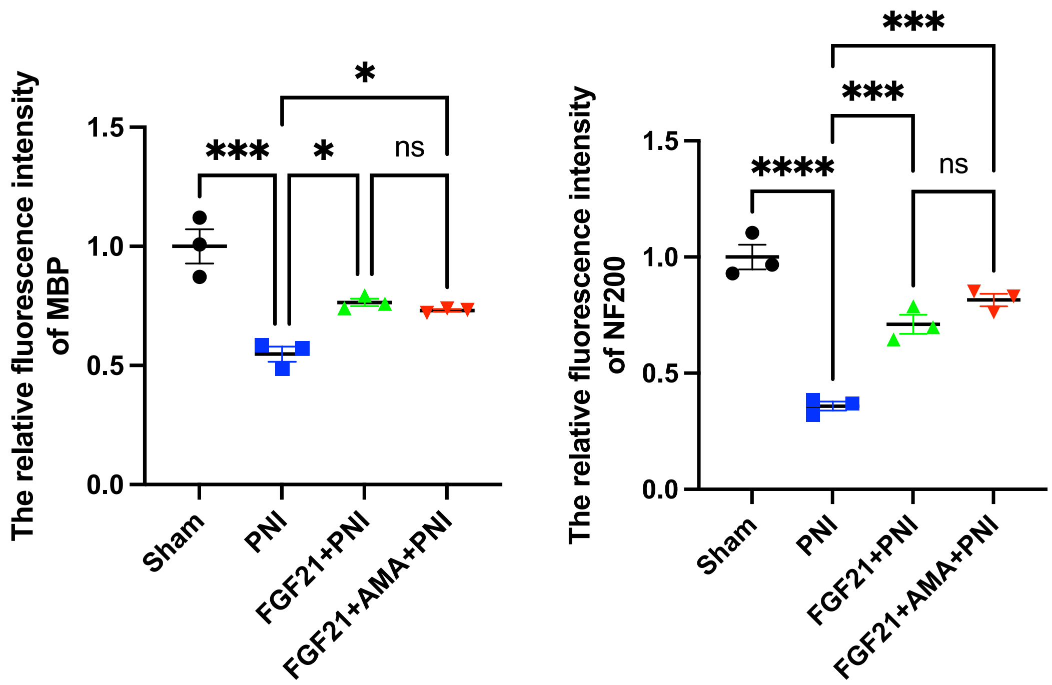

S Fig. 4 The fluorescence intensity of MBP and NF200 in Nerve per unit area.
